# Supplementary material for: Multi-proteomic profiling of the varicella-zoster virus–host interface reveals host susceptibilities to severe infection
Source: Nat Microbiol. 2025 Jul 30;10(8):2048–72. doi: 10.1038/s41564-025-02068-7 (PMC12313529; doi:10.1038/s41564-025-02068-7)
Supplement: Supplementary file 1 — Supplementary Discussion and References. [file 41564_2025_2068_MOESM1_ESM.pdf]

nature microbiology

Supplementary information

https://doi.org/10.1038/s41564-025-02068-7

Multi-proteomic profiling of the varicella-zoster virus–host interface reveals host susceptibilities to severe infection

In the format provided by the authors and unedited

Supplementary Discussion

Limitations of the study.

We focused our analysis on ORFs derived from the attenuated VZV rOka vaccine strain, which bears strain-specific mutations and is attenuated for growth in skin, as compared to other virus strains. Thus, interactions and effects specific to other VZV strains and to clinical isolates, or involving cellular proteins that may be expressed in other VZV target cells such as skin epidermis or T-cells, would not be identified in this study. However, the choice of a single syngeneic experimental design and a well-defined viral strain allowed us to project the activity of individual viral proteins on the cellular perturbations induced by VZV infections. While this setup is essential for meaningful data integration and for data mining of such a large resource, it is important to consider that some of the findings reported in this study may be neuron- or virus strain-specific. Moreover, the required C-terminal affinity tag of the viral proteins and the absence of potential viral binding partners can affect functional interactions[122]. Six VZV ORFs could not be included in this study (ORF2, -22, -27, -31, 42/45, -62). Two constructs (ORF22 and -42/45) could not be cloned, and we could not generate lentivirus for the other four.

Previously reported results supported SK-N-BE2 cells as a model to study VZV-host interactions.

In VZV-infected SK-N-BE2 cells (Figure 1b), we observed an up-regulation of proteins involved in cell division (MYCN and enrichment for components of the spindle apparatus). Several of these proteins (AURKA, ASPM, CCNB1, FAM83D, KIF11, CKS2) have previously been reported to be upregulated during productive VZV infection[23,123,124] and might be indicative of a promotion of late cell cycle progression, which is commonly observed in VZV-infected cells[125]. The downregulation of TP53 and the upregulation of BAG3 indicated an inhibition of apoptosis in VZV-infected SK-N-BE2 cells, validating previous reports of neuron-specific resistance to VZV-induced apoptosis[23,126,127]. The downregulation of the neuronal growth factor GAP43 was also reported during in vivo neuro-infections with HSV-1[128] and LCMV[129].

The overexpression of individual VZV ORFs in SK-N-BE2 cells recapitulated previously reported observations. We analysed by immunofluorescence the subcellular localisation of four VZV ORFs, which are representative of different cellular compartments (Extended Data Fig 2b). ORFs -9, -49, -63 and -67 were enriched in the cytosol, the Golgi apparatus and vesicles, the nucleus and the cell membrane, respectively, matching with their previously reported

subcellular localisation in infected cells[39–42]. Moreover, our analysis of the VZV-host protein-protein interactions confirmed known associations (Figure 2a, Extended Data Fig. 2e; Supplementary Table S2-2). For example, ORF63 was interacting with two ASF1 subunits[130], ORF4 with its mRNA export co-factors (NFX1, ALYREF, SRSF1, SRSF3 and SRSF7)[15] and ORF12 with the PI3K activator PIK3R1[113]. Cellular complexes enrichment analysis further highlighted previously reported interactions which are functional during infection (Figure 2b, Supplementary Table S2-3): the interaction of the phosphatidyl-inositol 3 kinase (PI3K) complex (including PIK3R1) with ORF12 (Tegument protein HSV-1 UL46 homologue), which functions to activate Akt signalling during infection[113]; the transcription export complex (including NFX1, ALYREF, THOCs) with ORF4 (mRNA export factor HSV-1 ICP27 homologue), which participates in the regulation of viral and host mRNA level[15]; the PTW/PP1 phosphatase complex (including PPP1CA/B) with ORF38, the latter being proposed to act as a phosphatase adaptor protein[131].

Supplementary discussion on the proteome changes in VZV-infected SK-N-BE2 cells.

The VZV infection of neuronal SK-N-BE2 cells triggered the deregulation of numerous proteins with known antiviral functions. For instance, we observed the decreased abundance the SMC5/6 complex. Interestingly, it is a known suppressor of viral transcription[132–135], and some viruses, including Hepatitis B Virus[136] and Epstein-Barr Virus (EBV)[133], engage host E3 ubiquitin ligases to mediate its proteasomal degradation. Several dysregulated proteins are involved in mounting the antiviral innate immune response or known viral restriction factors. IFI16 is a cofactor of viral DNA sensing[32] and a restriction factor of HSV-1[137]. hnRNPA2B1 was recently identified as a nuclear viral DNA sensor during HSV-1 infection[33]. The subunits of the RNA polymerase III complex (e.g. GTF3C1, -2, -3), are essential to mount an antiviral innate immune response to herpesviruses[34,138]. Besides proteins involved in DNA sensing, we observed strong downregulation of the 5'-3' RNA exoribonuclease XRN2 and its cofactor CDKN2AIPNL[139]. XRN2 is involved in the termination of cellular transcription[140] and viral RNA degradation[35]. Its dysregulation could contribute to the host transcriptional shut-off commonly employed by viruses to escape antiviral expression programs.

Detailed discussion of the ORF12 and ORF9 subnetworks obtained by network diffusion.

The ORF12 subnetwork is enriched for proteins that are in line with ORF12 functions (featured in Figure 4g, Extended Data Fig. 5 g). Its targeting of the PI3K complex (PIK3R1, PIK3R2, PIK3R3, PIK3CA, PIK3CB) has been shown to be of functional importance for VZV and drives Akt signalling[76]. Additionally, ORF12 associates with PI3K co-factors within the Receptor

Tyrosine Kinases (RTK) signalling complex (GRB2, GAB1, GAB2 and PTPN11), which was functionally connected to the upregulation of anti-apoptotic (BAG3, BCL2, DEPTOR, UNC5B) and downregulation of pro-apoptotic proteins (DAPK1, FADD, ENDOG), which corroborated the previously reported role of ORF12 to block apoptosis[141]. Concurrently, ORF12 increased the abundance of the protein kinase C-α (PRKCA), another effector of RTK signalling, which signals as a PI3K-dependent inhibitor of apoptosis[142], and downregulated several factors of G1/S transition (SKP2, CKS1B, CCND1, TAF1, RBM3) as well as DNA synthesis (DHFR, TYMS). The network diffusion analysis also revealed 106 intermediate proteins that connected ORF12 targets and their downstream effects (Extended Data Fig. 5g, Figure 4g). For example, the increased abundance of PRKCA correlated with the upregulation of BCL2 *via* the intermediate cyclic AMP-responsive element-binding protein (CREB1). CREB1 is a substrate of PRKCA[143] and its phosphorylation precedes activation of BCL2 transcription[144]. Importantly, CREB1 is phosphorylated in VZV-infected cells, where this was shown to be relevant for viral replication[145]. ORF9 is a tegument protein which binds to the cytoskeletal network during virion assembly and egress[72,73], and, thus, is essential for viral replication[74]. Specifically, target-effect connections between proteins involved in cytoskeleton organisation were observed (Figure 4h). ORF9 upregulated centrin-2 (CETN2), which is a central regulator of cytoskeleton components that were detected as binders of ORF9 (microtubule-associated proteins TUBB, TUBB4A, TUBB4B, ACTR1A, CEP131, CEP78; the dynactin complex DCTN1, DCTN2, DCTN3; ciliary basal body components NPHP4, RPGRIP1L). Interestingly, network diffusion highlighted two ORF9-bound complexes that are known to regulate CETN2: the UV-DDB-Ubiquitin ligase complex comprising DDB1, RBX1/ROC1 and the COP9 signalosome, which recruits CETN2 on one of its functional sites[146], and the CBX4-PCGF2-PHC2 polycomb complex that SUMOylates CETN2[147]. These interactions between ORF9 and regulatory complexes may be valuable targets for therapeutic approaches aiming at limiting ORF9 function and thereby VZV replication.

Discussion on the host gene knockout screen readout and the identified functional factors.

Discussion on the correction for the variability of cell density at infection: The co-culture of target-knockout (BFP) and control cells (GFP) allows accounting for the variability of cell density at infection, which is inherent to large array cellular screens and which we identified as a strong bias to determine relative VZV propagation. Specifically, this allows intra-well normalisation by calculating the target-to-control MRI ratio per well. The comparison of the MRI, average or ratio across BFP and GFP populations, with the cell density (displayed as

absolute difference to the median luminescence intensity) showed that extremely low or high confluences correlate with reduced VZV propagation (Figure S5A, left). This might be explained by a reduced cell-to-cell contact across a low-density cell layer, and reduced cellular activity in confluent cells. The target-to-control MRI ratio, however, was independent of cell density at infection (Figure S5A, right) and then allowed unbiased relative evaluation of each knockout's effects.

Detailed discussion on the identified host factors and the cellular functions involved: Host proteins with profound and unexpected antiviral functions were MTERF3 (mitochondrial transcription), ABR (neuronal axon branching), KLHL12 (WNT and secretory pathways) and ASF1A (epigenetic regulation). Among proteins with proviral functions were ING1 (apoptosis), GRAMD1A (cholesterol transport), PUSL1 (mitochondrial translation), PIK3CA (PI3K signalling), MPP8 (epigenetic silencing), ZNF280D (transcription) and HGF (MET signalling).

The function of MPP8 as proviral is unexpected as the protein has been reported as part of viral restriction mechanism *via* the silencing by the HUSH complex of viral expression. However, the HUSH complex has also been reported to repress regions of the human genome encoding antiviral molecules[148,149]. The detected interactions of ORF12 and ORF4 with the HUSH components (MPP8, TASOR and PPHLN1) (Figure 2a) might mediate the sequestration of the complex onto the host genome to prevent expression of antiviral factors.

The potent binding of ZNF280D to the VZV transcriptional regulator ORF63 (Figure 2a) might participate in a hijacking mechanism by the viral transcription machinery.

Detailed discussion on multi-level regulations of key cellular pathways by VZV.

An example of the complexity of herpesvirus signalling is the crosstalk between apoptosis and cell cycle regulation in different infected tissues. Herpesvirus infection, including VZV, induces necroptosis or apoptosis in skin, lung and immune cells, while cell death induction is prevented in neurons[23,126,127]. In line with this notion, our proteomic profiling of VZV-infected SK-N-BE2 neuroblastoma cells showed an anti-apoptotic signature (Figure 1b). It was proposed that ORF12 impairs cell death induction through the MEK-ERK pathway and in a PI3K-independent manner in fibroblasts[141,150]. Combined analysis of interactome and effectome data using network diffusion suggested that components of the RTK signalling complex (PI3K, GRB2, GAB1/2, PTPN11 and PRKCA) are key mediators of the ORF12 anti-apoptotic function. It may explain the activation of MEK-ERK and suggests that this ORF12-

dependent anti-apoptotic mechanism is shared across cell lineages (Figure 4g). Concurrent to apoptosis inhibition, our data indicated that the late phase of the cell cycle is activated in VZV-infected SK-N-BE2 cells (Figure 1b), as published for VZV-infected human foreskin fibroblasts, epithelial cells and stem cell-derived neurons[23,24,125]. ORF12 was also reported to mediate this activation through interaction and activation of the PI3K-Akt axis in MeWo cells[113]. However, while our data confirmed the strong association of ORF12 to the PI3K components in SK-N-BE2 cells, it did not recapitulate the increased expression of G2/M cyclins in ORF12-expressing cells. In contrast, our analysis suggests that ORF12 expression paused early cell cycle progression and DNA replication by downregulating activators of the G1/S - phase and DNA synthesis. Thus, while the cell cycle is generally activated during VZV infection, our data suggest cell type-specific downstream consequences of the engagement of the PI3K complex by ORF12 in neuroblastoma or fibroblast cells. Our data contained several other viral ORFs that regulated cell cycle progression, such as ORF7, which downregulated proteins of the G2/M transition, and ORF8, which reduced the abundance of the G2/M CCNB1-CDK1 complex (Figure 3b). It is possible that these viral proteins are temporally involved in cell cycle regulation during the viral replication phase, and that their effects are further tuned by other mechanisms. For instance, we detected that ORF4 and ORF9 bind to the anaphase-promoting complex and thus might favour the accumulation of cyclins and cell cycle progression (Figure 2a). In addition, ORF7, ORF33.5 and ORF66 triggered the downregulation of p53, which was also observed in infected cells (Figure 1b, 3b). These effects might coordinately limit cell cycle arrest and lead to the anti-apoptotic signature observed in virus-infected cells.

Similarly, the REST response is differentially regulated by ORF66, ORF9A and ORF38 (Figure 3b). Since all three proteins are predominantly transcribed at the late phase of infections[88], yet undefined regulatory denominators such as cellular homeostasis and metabolism may mask the activity of one or the other protein in the context of VZV infections. Altogether, time-dependent expression of individual viral proteins, which is particularly prominent for DNA-viruses[88], as well as regulations of the cellular environment, may be obscured in a population of non-synchronously infected cells. Thus, profiling of effects elicited by the expression of individual ORF may reveal in-depth insights into functions which are more difficult to detect in populations of infected cells and points towards ORF-targeted experiments in VZV-infected conditions.

This profiling of the VZV-host interface reflects specific and general herpesvirus functions.

The host DNA damage response (DDR) recognises double-strand breaks (DSB) on the DNA virus genome and is strongly involved in controlling its replication. Early after viral entry, the non-homologous end-joining (NHEJ) complex targets the viral genome and restricts its replication[151,152]. Yet, viruses have evolved escape mechanisms against the NHEJ complex: adenovirus degrades LIG4, and the HSV-1 E3 ubiquitin ligase ICP0 targets PRKDC and XCRR5[153–155]. During the late phase of infection, each herpesvirus recruits a unique set of DDR factors to replication compartments (RC) to initiate recombination-dependent replication (reviewed in [156]). The HSV-1 replicase UL12 hijacks the homology directed repair (HDR) pathway by binding to the MRN complex (RAD50, MRE11, NSB1), which initiates the recruitment of ATR and ATRIP[157,158]. KSHV induces the recruitment of both MRN (HDR) and XRCC5/6 (NHEJ) complexes at RC, but not ATR[151]. Notably, HDR and NHEJ proteins have pro- and antiviral activity, respectively. VZV infection activates host DDR, yet the host factors involved remain unknown[159]. Here, we observed that the VZV UL12 homologue, ORF48, strongly engages the NHEJ complex (LIG4, PAXX, APLF, NHEJ1, XRCC4, XRCC6, PRKDC) and to some extent RAD50, but not ATR (Figure 2a and 2b). Moreover, degradation of NHEJ proteins, as reported for other viruses, was not apparent in the proteome of VZV-infected or ORF-expressing SK-N-BE2 cells. Unlike HSV-1, VZV thus employs specific molecular mechanisms to hijack the host DDR via the recruitment of the NHEJ complex by ORF48.

Chromatin remodelling modulates the accessibility of the host and viral genome for transcription, and its regulation seems to be highly similar between HSV-1 and VZV. Along this line, chromatin silencers involved in DNA virus restriction were downregulated in VZV-infected SK-N-BE2 cells (Figure 1b), as previously reported in other models[132,133]. Moreover, similarly to their HSV-1 homologues, ORF63 (ICP22)[160] and ORF9 (VP22)[161] interacted with chromatin regulators (Figure 2a). Extending the picture, we further identified the primase-associated factor ORF52 and the thymidine kinase ORF36 as prominent interactors of the polycomb complex (Figure 2), which is involved in herpesvirus latency establishment[162]. While the SK-N-BE2 infection model is not designed to recapitulate viral functions associated to latency, we expect that the individual viral proteins retain interactions when expressed in this cell line.

A pan-herpesvirus target to limit the antiviral response and to promote viral genome replication and transcription is the host gene expression machinery. The mediator complex connects transcriptional factors, which bind specific DNA regions, to the catalytic RNA polymerase II

(POLR2). Thus, it constitutes an appropriate target for viruses to redirect the activity of POLR2 to transcriptional regions of their own interest. HSV-1 VP16 interacts with the mediator subunit MED25 shortly after viral entry, to transactivate viral immediate-early (IE) genes transcription[163]. VZV ORF62, the major transcriptional regulator of all viral genes, also functions through binding to MED25[12]. Here, we identified that the VZV homologue of VP16, ORF10, similarly recruits the mediator complex, most likely to initiate transcription of viral IE genes (Figure 2). Also, our study unveiled an interaction between ORF32, which does not have a homologue in other herpesviruses, and the RNA polymerase II co-factor GTF2B (Figure 2a, Extended Data Fig. 2f-h). GTF2B is required for *de novo* initiation of gene transcription, which is an essential step for the expression of many antiviral and inflammatory genes, and also targeted by other viruses[118]. Thus, ORF32 might be involved in the regulation of GTF2B to favour viral replication, suggesting a VZV-specific mechanism of host gene expression manipulation.

The role in virion release of the cytoplasmic envelopment protein 1, encoded by VZV ORF53, is conserved across herpesviruses[164–167]. The virion is released in the Trans-Golgi Network (TGN)[168,169] - however, no host co-factor has yet been identified to be involved in this process. We detected interactions between ORF53 and the guanine nucleotide exchange factors DENND4A and DENND4C (Figure 2a), the members of the DENN-containing protein family that can be directed to intracellular membranes via their C-terminal MABP domain[164–167,170]. Notably, DENND4C associates with the Ras-Related GTP-binding protein RAB10[54], which has been recently identified as a factor of Epstein-Barr-Virus egress through virion association in the TGN[55]. Our data suggest that herpesvirus may, *via* their conserved encapsidation proteins, associate with the host TGN exocytosis machinery in the course of secondary envelopment.

Herpesviruses generally target the innate immune system, which is evidenced by the absence of ISG and pro-inflammatory gene expression following VZV infection (Figure 1b) and binding of several viral proteins to key factors of the innate immune response (Figure 2a). Despite this lack of antiviral response, alphaherpesviruses have recently been shown to be sensitive to type-I interferon treatment in neurons[171,172], and VZV to IL-6[173]. Our analysis of the VZV-host relationship links these findings with antiviral evasion strategies. We identified several proteins that are involved in the escape of viral sensing by the host and thus limit activation of the endogenous type-I interferon response. We identified the VCP-UBXN7 complex, a regulator of host E3 ubiquitin ligase activity, as an interactor of ORF61 during infection (Figure 4d) and a co-factor of ORF61-dependant IFI16 degradation (Figure 4e). Degradation of IFI16 has also

been reported for the HSV-1 homologue ICP0[66]. We hypothesise that herpesviruses may generally engage this complex to degrade IFI16. A notable example for the perturbation of the intracellular VZV-specific sensing machinery involved the GTF3C complex. Its components are essential cofactors of RNA polymerase III, which acts as a sensor of the VZV AT-rich DNA and drives the type-I interferon response[119,174]. GTF3C components are downregulated during infection of SK-N-BE2 cells (Figure 1b) and are targeted by ORF9 (Figure 2). However, the expression of ORF9 itself does not alter the expression of the complex, indicating engagement of additional processes in the context of virus infection. The NF-κB pathway is hijacked by herpesviruses to escape the pro-inflammatory response concurrently to the promotion of viral transcription[175,176]. Both HSV-1 and VZV are reported to target NF-κB factors and alter the downstream response via their E3 ubiquitin ligase ICP0/ORF61 and their kinase US3/ORF66[177–179]. ORF61, in particular, induces the cytoplasmic sequestration of the NF-κB transcription factors p50/p65[180] by blocking the ubiquitination and degradation of IκBα[177]. Our study identified the NF-κB regulator USP11 as cellular partner of ORF61 and ORF66, and NKIRAS2, an inhibitor of the inflammatory pathway, as binding partner of ORF66 (Figure 2a). Interestingly, USP11 is a deubiquitinase which was shown to prevent NF-κB activation *via* stabilisation of IκBα in the cytoplasm[181]. Moreover, as its activity strongly depends on its phosphorylation status[182], ORF66-mediated USP11 phosphorylation may stabilise IκBα to limit NF-κB signalling in VZV infected cells. Thus, the here generated VZV interactomes provide potential molecular mechanisms for NF-κB regulation by VZV and HSV-1.

The reactivation of both VZV and HSV-1 was recently associated with neurodegenerative diseases, including multiple sclerosis[6]. Here, we observed that neuronal differentiation and development is repressed in infected SK-N-BE2 cells (Figure 1c) as it was previously demonstrated after HSV-1 reactivation in latently infected neurons *in vivo*[128]. Particularly interesting in VZV-infected cells is the downregulation of Neuromodulin (GAP43), which has also been reported to be decreased in patients with multiple sclerosis, possibly leading to axonal regeneration defects[183,184]. Notably, ORF38 expression is sufficient to degrade GAP43 (Extended Data Fig. 5a), and may thus serve as a viral protein contributing to neuronal pathogenicity. Importantly, GAP43 contains multiple phosphorylation sites regulating its activity and cellular localisation[185,186]. The ORF38 interactome contained the protein phosphatase PP1 (Figure 2a), as has also been shown for its HSV-1 homologue UL21[131], which could potentially be involved in GAP43 phosphorylation and be a cellular target to modulate GAP43. Further investigation of the effects of GAP43 phosphorylation on its abundance and

the involvement of ORF38/UL21 might guide the use of phosphatase activity inhibitors to limit viral neuronal pathogenicity.

Insights into the antiviral function of NPHP4 and the association of its variant S862N with VZV pathogenicity.

NPHP4 (binding ORF4, -9 and antiviral) (Figure 2a, Figure 5c) is mutated in a patient presenting with VZV-associated meningoencephalitis (Figure 6a). We found that the patient mutation encoding for NPHP4 S862N impaired its anti-VZV activity (Figure 6c). Moreover, it is part of a 14-3-3 recognition motif ($RX_{1-2}SX_{2-3}S$), which explains the selective loss of interactions with 14-3-3ε (YWHAE) and 14-3-3η (YWHAH) (Figure 6d). 14-3-3 proteins are regulatory factors involved in a plethora of cellular and viral functions. They have prominent roles in the life cycle of several herpesviruses, including in the latency-reactivation cycle (KSHV, EBV), in apoptosis and neuroinvasion, and in intracellular trafficking (HSV-1 and -2) (reviewed in [187]). Also, 14-3-3 proteins are involved in the mounting of the viral RNA sensing machinery, which is relevant for herpesviruses[188,189]. While NPHP4 is mostly characterised as a scaffold protein of the cilia transition zone *via* genetic variants associated with ciliopathies[190,191], which is in line with the binding of NPHP4 with the cilia proteins (Figure 6d). However, the similar association of cilia proteins to NPHP4 with the patient mutation indicates that this function is likely not involved in defence against VZV. NPHP4 also represses WNT activation by promoting the translocation of the β-catenin inhibitor JADE1[192]. Our transcriptome data of NPHP4-depleted and VZV-infected cells suggests that WNT signalling is induced during infection and repressed by NPHP4. Notably, 14-3-3 proteins were reported to function in the vicinity of NPHP4 in a mechanism that controls the translocation and activation of YAP-TAZ, yet no direct binding was described[193]. Our approach identified an interaction of NPHP4 with 14-3-3 proteins and associates this function with VZV restriction. Follow-up studies are required to dissect the exact implication of the NPHP4-14-3-3 binding in the defence against VZV and how the patient variant disrupting this interaction can translate into disease.

Supplementary references

122. Uetz, P. *et al.* Herpesviral protein networks and their interaction with the human proteome. *Science* **311**, 239–242 (2006).

123. Ouwendijk, W. J. D. *et al.* Analysis of Virus and Host Proteomes During Productive HSV-1 and VZV Infection in Human Epithelial Cells. *Front. Microbiol.* **11**, 1179 (2020).
124. Leisenfelder, S. A., Kinchington, P. R. & Moffat, J. F. Cyclin-dependent kinase 1/cyclin B1 phosphorylates varicella-zoster virus IE62 and is incorporated into virions. *J. Virol.* **82**, 12116–12125 (2008).
125. Leisenfelder, S. A. & Moffat, J. F. Varicella-zoster virus infection of human foreskin fibroblast cells results in atypical cyclin expression and cyclin-dependent kinase activity. *J. Virol.* **80**, 5577–5587 (2006).
126. Hood, C., Cunningham, A. L., Slobedman, B., Boadle, R. A. & Abendroth, A. Varicella-zoster virus-infected human sensory neurons are resistant to apoptosis, yet human foreskin fibroblasts are susceptible: evidence for a cell-type-specific apoptotic response. *J. Virol.* **77**, 12852–12864 (2003).
127. Kennedy, P. G. *et al.* Varicella-Zoster Virus infected human neurons are resistant to apoptosis. *J. Neurovirol.* **26**, 330–337 (2020).
128. Martin, R. E., Henken, D. B. & Hill, J. M. Altered expression and changing distribution of the nerve growth associated protein GAP-43 during ocular HSV-1 infection in the rabbit. *J. Neurovirol.* **2**, 127–135 (1996).
129. de la Torre, J. C. *et al.* Viral persistence in neurons alters synaptic plasticity and cognitive functions without destruction of brain cells. *Virology* **220**, 508–515 (1996).
130. Ambagala, A. P. *et al.* Varicella-zoster virus immediate-early 63 protein interacts with human antisilencing function 1 protein and alters its ability to bind histones h3.1 and h3.3. *J. Virol.* **83**, 200–209 (2009).
131. Benedyk, T. H. *et al.* pUL21 is a viral phosphatase adaptor that promotes herpes simplex virus replication and spread. *PLoS Pathog.* **17**, e1009824 (2021).
132. Xu, W. *et al.* PJA1 Coordinates with the SMC5/6 Complex To Restrict DNA Viruses and Episomal Genes in an Interferon-Independent Manner. *J. Virol.* **92**, e00825-18 (2018).

133. Yiu, S. P. T., Guo, R., Zerbe, C., Weekes, M. P. & Gewurz, B. E. Epstein-Barr virus BNRF1 destabilizes SMC5/6 cohesin complexes to evade its restriction of replication compartments. *Cell Rep.* **38**, 110411 (2022).

134. Dupont, L. *et al.* The SMC5/6 complex compacts and silences unintegrated HIV-1 DNA and is antagonized by Vpr. *Cell Host Microbe* **29**, 792-805.e6 (2021).

135. Gibson, R. T. & Androphy, E. J. The SMC5/6 Complex Represses the Replicative Program of High-Risk Human Papillomavirus Type 31. *Pathog. Basel Switz.* **9**, E786 (2020).

136. Murphy, C. M. *et al.* Hepatitis B Virus X Protein Promotes Degradation of SMC5/6 to Enhance HBV Replication. *Cell Rep.* **16**, 2846–2854 (2016).

137. Orzalli, M. H., Conwell, S. E., Berrios, C., DeCaprio, J. A. & Knipe, D. M. Nuclear interferon-inducible protein 16 promotes silencing of herpesviral and transfected DNA. *Proc. Natl. Acad. Sci. U. S. A.* **110**, E4492-4501 (2013).

138. Naesens, L. *et al.* GTF3A mutations predispose to herpes simplex encephalitis by disrupting biogenesis of the host-derived RIG-I ligand RNA5SP141. *Sci. Immunol.* **7**, eabq4531 (2022).

139. Richter, H., Katic, I., Gut, H. & Großhans, H. Structural basis and function of XRN2 binding by XTB domains. *Nat. Struct. Mol. Biol.* **23**, 164–171 (2016).

140. West, S., Gromak, N. & Proudfoot, N. J. Human 5’ --> 3’ exonuclease Xrn2 promotes transcription termination at co-transcriptional cleavage sites. *Nature* **432**, 522–525 (2004).

141. Liu, X., Li, Q., Dowdell, K., Fischer, E. R. & Cohen, J. I. Varicella-Zoster virus ORF12 protein triggers phosphorylation of ERK1/2 and inhibits apoptosis. *J. Virol.* **86**, 3143–3151 (2012).

142. Li, W. *et al.* Protein kinase C-alpha overexpression stimulates Akt activity and suppresses apoptosis induced by interleukin 3 withdrawal. *Oncogene* **18**, 6564–6572 (1999).

143. Martín, F., Mora, L., Laorden, M. & Milanés, M. Protein kinase C phosphorylates the cAMP response element binding protein in the hypothalamic paraventricular nucleus during morphine withdrawal. *Br. J. Pharmacol.* **163**, 857–875 (2011).

144. Wilson, B. E., Mochon, E. & Boxer, L. M. Induction of bcl-2 expression by phosphorylated CREB proteins during B-cell activation and rescue from apoptosis. *Mol. Cell. Biol.* **16**, 5546–5556 (1996).

145. François, S. *et al.* Varicella-Zoster Virus Activates CREB, and Inhibition of the pCREB-p300/CBP Interaction Inhibits Viral Replication In Vitro and Skin Pathogenesis In Vivo. *J. Virol.* **90**, 8686–8697 (2016).

146. Sugasawa, K. *et al.* UV-induced ubiquitylation of XPC protein mediated by UV-DDB-ubiquitin ligase complex. *Cell* **121**, 387–400 (2005).

147. Klein, U. R. & Nigg, E. A. SUMO-dependent regulation of centrin-2. *J. Cell Sci.* **122**, 3312–3321 (2009).

148. Liu, N. *et al.* Selective silencing of euchromatic L1s revealed by genome-wide screens for L1 regulators. *Nature* **553**, 228–232 (2018).

149. Tunbak, H. *et al.* The HUSH complex is a gatekeeper of type I interferon through epigenetic regulation of LINE-1s. *Nat. Commun.* **11**, 5387 (2020).

150. Liu, X. & Cohen, J. I. Inhibition of Bim enhances replication of varicella-zoster virus and delays plaque formation in virus-infected cells. *J. Virol.* **88**, 1381–1388 (2014).

151. Hollingworth, R., Horniblow, R. D., Forrest, C., Stewart, G. S. & Grand, R. J. Localization of Double-Strand Break Repair Proteins to Viral Replication Compartments following Lytic Reactivation of Kaposi's Sarcoma-Associated Herpesvirus. *J. Virol.* **91**, e00930-17 (2017).

152. Taylor, T. J. & Knipe, D. M. Proteomics of herpes simplex virus replication compartments: association of cellular DNA replication, repair, recombination, and chromatin remodeling proteins with ICP8. *J. Virol.* **78**, 5856–5866 (2004).

153. Baker, A., Rohleder, K. J., Hanakahi, L. A. & Ketner, G. Adenovirus E4 34k and E1b 55k oncoproteins target host DNA ligase IV for proteasomal degradation. *J. Virol.* **81**, 7034–7040 (2007).

154. Parkinson, J., Lees-Miller, S. P. & Everett, R. D. Herpes simplex virus type 1 immediate-early protein vmw110 induces the proteasome-dependent degradation of the catalytic subunit of DNA-dependent protein kinase. *J. Virol.* **73**, 650–657 (1999).

155. De Chiara, G. *et al.* Herpes Simplex Virus-Type1 (HSV-1) Impairs DNA Repair in Cortical Neurons. *Front. Aging Neurosci.* **8**, 242 (2016).

156. Full, F. & Ensser, A. Early Nuclear Events after Herpesviral Infection. *J. Clin. Med.* **8**, 1408 (2019).

157. Lilley, C. E., Carson, C. T., Muotri, A. R., Gage, F. H. & Weitzman, M. D. DNA repair proteins affect the lifecycle of herpes simplex virus 1. *Proc. Natl. Acad. Sci. U. S. A.* **102**, 5844–5849 (2005).

158. Mohni, K. N., Livingston, C. M., Cortez, D. & Weller, S. K. ATR and ATRIP are recruited to herpes simplex virus type 1 replication compartments even though ATR signaling is disabled. *J. Virol.* **84**, 12152–12164 (2010).

159. Yamamoto, T., Ali, M. A., Liu, X. & Cohen, J. I. Activation of H2AX and ATM in varicella-zoster virus (VZV)-infected cells is associated with expression of specific VZV genes. *Virology* **452–453**, 52–58 (2014).

160. Fox, H. L., Dembowski, J. A. & DeLuca, N. A. A Herpesviral Immediate Early Protein Promotes Transcription Elongation of Viral Transcripts. *mBio* **8**, e00745-17 (2017).

161. López, M. R., Schlegel, E. F. M., Wintersteller, S. & Blaho, J. A. The major tegument structural protein VP22 targets areas of dispersed nucleolin and marginalized chromatin during productive herpes simplex virus 1 infection. *Virus Res.* **136**, 175–188 (2008).

162. Kwiatkowski, D. L., Thompson, H. W. & Bloom, D. C. The polycomb group protein Bmi1 binds to the herpes simplex virus 1 latent genome and maintains repressive histone marks during latency. *J. Virol.* **83**, 8173–8181 (2009).

163. Yang, F., DeBeaumont, R., Zhou, S. & Näär, A. M. The activator-recruited cofactor/Mediator coactivator subunit ARC92 is a functionally important target of the VP16 transcriptional activator. *Proc. Natl. Acad. Sci. U. S. A.* **101**, 2339–2344 (2004).

164. Schmitt, J. & Keil, G. M. Identification and characterization of the bovine herpesvirus 1 UL7 gene and gene product which are not essential for virus replication in cell culture. *J. Virol.* **70**, 1091–1099 (1996).

165. Fuchs, W. *et al.* The UL7 gene of pseudorabies virus encodes a nonessential structural protein which is involved in virion formation and egress. *J. Virol.* **79**, 11291–11299 (2005).

166. He, H.-P. *et al.* Structure of Epstein-Barr virus tegument protein complex BBRF2-BSRF1 reveals its potential role in viral envelopment. *Nat. Commun.* **11**, 5405 (2020).

167. Mocarski Jr., E. S. Comparative analysis of herpesvirus-common proteins. in *Human Herpesviruses: Biology, Therapy, and Immunoprophylaxis* (eds. Arvin, A. et al.) (Cambridge University Press, Cambridge, 2007).

168. Butt, B. G. *et al.* Insights into herpesvirus assembly from the structure of the pUL7:pUL51 complex. *eLife* **9**, e53789 (2020).

169. Wang, W. *et al.* Varicella-zoster virus ORF7 interacts with ORF53 and plays a role in its trans-Golgi network localization. *Virol. Sin.* **32**, 387–395 (2017).

170. Boura, E. & Hurley, J. H. Structural basis for membrane targeting by the MVB12-associated β-prism domain of the human ESCRT-I MVB12 subunit. *Proc. Natl. Acad. Sci. U. S. A.* **109**, 1901–1906 (2012).

171. Song, R. *et al.* Two Modes of the Axonal Interferon Response Limit Alphaherpesvirus Neuroinvasion. *mBio* **7**, e02145-02115 (2016).

172. Boeren, M. *et al.* Activation of Interferon-Stimulated Genes following Varicella-Zoster Virus Infection in a Human iPSC-Derived Neuronal In Vitro Model Depends on Exogenous Interferon-α. *Viruses* **14**, 2517 (2022).

173. Como, C. N., Pearce, C. M., Cohrs, R. J. & Baird, N. L. Interleukin-6 and type 1 interferons inhibit varicella zoster virus replication in human neurons. *Virology* **522**, 13–18 (2018).

174. Ogunjimi, B. *et al.* Inborn errors in RNA polymerase III underlie severe varicella zoster virus infections. *J. Clin. Invest.* **127**, 3543–3556 (2017).

175. Amici, C. *et al.* Herpes simplex virus disrupts NF-kappaB regulation by blocking its recruitment on the IkappaBalpha promoter and directing the factor on viral genes. *J. Biol. Chem.* **281**, 7110–7117 (2006).
176. El Mjiyad, N. *et al.* Varicella-zoster virus modulates NF-kappaB recruitment on selected cellular promoters. *J. Virol.* **81**, 13092–13104 (2007).
177. Whitmer, T. *et al.* The ORF61 Protein Encoded by Simian Varicella Virus and Varicella-Zoster Virus Inhibits NF-κB Signaling by Interfering with IκBα Degradation. *J. Virol.* **89**, 8687–8700 (2015).
178. Wang, K., Ni, L., Wang, S. & Zheng, C. Herpes simplex virus 1 protein kinase US3 hyperphosphorylates p65/RelA and dampens NF-κB activation. *J. Virol.* **88**, 7941–7951 (2014).
179. Daubeuf, S. *et al.* HSV ICP0 recruits USP7 to modulate TLR-mediated innate response. *Blood* **113**, 3264–3275 (2009).
180. Jones, J. O. & Arvin, A. M. Inhibition of the NF-kappaB pathway by varicella-zoster virus in vitro and in human epidermal cells in vivo. *J. Virol.* **80**, 5113–5124 (2006).
181. Sun, W. *et al.* USP11 negatively regulates TNFalpha-induced NF-kappaB activation by targeting on IkappaBalpha. *Cell. Signal.* **22**, 386–394 (2010).
182. Kapadia, B. *et al.* Fatty Acid Synthase induced S6Kinase facilitates USP11-eIF4B complex formation for sustained oncogenic translation in DLBCL. *Nat. Commun.* **9**, 829 (2018).
183. Teunissen, C. E. *et al.* Growth-associated protein 43 in lesions and cerebrospinal fluid in multiple sclerosis. *Neuropathol. Appl. Neurobiol.* **32**, 318–331 (2006).
184. Sandelius, Å. *et al.* Cerebrospinal fluid growth-associated protein 43 in multiple sclerosis. *Sci. Rep.* **9**, 17309 (2019).
185. Wang, C.-Y. *et al.* Protein kinase C-dependent growth-associated protein 43 phosphorylation regulates gephyrin aggregation at developing GABAergic synapses. *Mol. Cell. Biol.* **35**, 1712–1726 (2015).

186. Gauthier-Kemper, A. *et al.* Interplay between phosphorylation and palmitoylation mediates plasma membrane targeting and sorting of GAP43. *Mol. Biol. Cell* **25**, 3284–3299 (2014).
187. Liu, J. *et al.* The role of 14-3-3 proteins in cell signalling pathways and virus infection. *J. Cell. Mol. Med.* **25**, 4173–4182 (2021).
188. Melchjorsen, J. *et al.* Early innate recognition of herpes simplex virus in human primary macrophages is mediated via the MDA5/MAVS-dependent and MDA5/MAVS/RNA polymerase III-independent pathways. *J. Virol.* **84**, 11350–11358 (2010).
189. Liu, H. M. *et al.* The Mitochondrial Targeting Chaperone 14-3-3ε Regulates a RIG-I Translocon that Mediates Membrane Association and Innate Antiviral Immunity. *Cell Host Microbe* **11**, 528–537 (2012).
190. Sang, L. *et al.* Mapping the NPHP-JBTS-MKS protein network reveals ciliopathy disease genes and pathways. *Cell* **145**, 513–528 (2011).
191. Schuermann, M. J. *et al.* Mapping of gene loci for nephronophthisis type 4 and Senior-Løken syndrome, to chromosome 1p36. *Am. J. Hum. Genet.* **70**, 1240–1246 (2002).
192. Burcklé, C. *et al.* Control of the Wnt pathways by nephrocystin-4 is required for morphogenesis of the zebrafish pronephros. *Hum. Mol. Genet.* **20**, 2611–2627 (2011).
193. Habbig, S. *et al.* NPHP4, a cilia-associated protein, negatively regulates the Hippo pathway. *J. Cell Biol.* **193**, 633–642 (2011).
