# Supplementary material for: Multi-proteomic profiling of the varicella-zoster virus–host interface reveals host susceptibilities to severe infection
Source: Nat Microbiol. 2025 Jul 30;10(8):2048–72. doi: 10.1038/s41564-025-02068-7 (PMC12313529; doi:10.1038/s41564-025-02068-7)
Supplement: Supplementary file 2 — Reporting Summary [file 41564_2025_2068_MOESM2_ESM.pdf]

nature portfolio

Corresponding author(s): Andreas Pichlmair

Last updated by author(s): Jun 13, 2025

Reporting Summary

Nature Portfolio wishes to improve the reproducibility of the work that we publish. This form provides structure for consistency and transparency in reporting. For further information on Nature Portfolio policies, see our Editorial Policies and the Editorial Policy Checklist.

Statistics

For all statistical analyses, confirm that the following items are present in the figure legend, table legend, main text, or Methods section.

n/a	Confirmed	
☐	☒	The exact sample size (*n*) for each experimental group/condition, given as a discrete number and unit of measurement
☐	☒	A statement on whether measurements were taken from distinct samples or whether the same sample was measured repeatedly
☐	☒	The statistical test(s) used AND whether they are one- or two-sided<br>*Only common tests should be described solely by name; describe more complex techniques in the Methods section.*
☐	☒	A description of all covariates tested
☐	☒	A description of any assumptions or corrections, such as tests of normality and adjustment for multiple comparisons
☐	☒	A full description of the statistical parameters including central tendency (e.g. means) or other basic estimates (e.g. regression coefficient) AND variation (e.g. standard deviation) or associated estimates of uncertainty (e.g. confidence intervals)
☐	☒	For null hypothesis testing, the test statistic (e.g. *F*, *t*, *r*) with confidence intervals, effect sizes, degrees of freedom and *P* value noted<br>*Give P values as exact values whenever suitable.*
☐	☒	For Bayesian analysis, information on the choice of priors and Markov chain Monte Carlo settings
☐	☒	For hierarchical and complex designs, identification of the appropriate level for tests and full reporting of outcomes
☐	☒	Estimates of effect sizes (e.g. Cohen's *d*, Pearson's *r*), indicating how they were calculated

Our web collection on statistics for biologists contains articles on many of the points above.

Software and code

Policy information about availability of computer code

Data collection

The mass spectrometer was operated and MS spectra acquired using the XCalibur software (Thermo Fisher Scientific).
Flow cytometry data: CytExpert software operating the CytoFLEX device.
Confocal images: Zen 3.5 (blue edition, ZEISS).
Live imaging data: IncuCyte software (v2021, Sartorius)
Transcriptome data: Illumina Nextseq 500 system; Genome mapping was done with Dropseq (v1.12, http://mccarrolllab.org/dropseq/)
WES data: Illumina Nextseq 550 system. GATK and BWA were used for alignment, calling variants and generate the VCF files.

Data analysis

MS data analysis: MaxQuant (v.1.6.0.15, 1.6.10.0, 1.6.14.0); Perseus (v.1.6.5.0, 2.0.7.0); R (v.3.6 and 4.1), R Studio (v.2022.07.2+576), Julia (v.1.5 and 1.6) and Python (v.3.8); In-house R and Julia packages and scripts used for the bioinformatics analysis of the data have been deposited to public GitHub repositories: in-house scripts (for msglm and hotnet): https://doi.org/10.5281/zenodo.15647702; msimportr: https://doi.org/10.5281/zenodo.7746897; msglm: https://doi.org/10.5281/zenodo.4536605, https://doi.org/10.5281/zenodo.4536604; gene set enrichment: https://doi.org/10.5281/zenodo.4536596; hierarchical hotnet: https://doi.org/10.5281/zenodo.10487870; rstan package (v.2.19).
Transcriptome analysis: DESeq2 (v.1.38.1)
Networks visualization: yEd (v.3.2)
FACS data analysis: FlowJo (version 10).
Western blot quantification: ImageLab (v. 6.0.1)
Live-cell images were processed and masked with IncuCyte Software; Sartorius, v2019B Rev2 or v2021).
Confocal images were processed with Zen blue edition (ZEISS): versions 3.5 (airyscan) and 3.8 (brightnes and contrast adjustments and intensity line profiles). Masking and quantification of confocal images were done with FIJI (ImageJ; version 1.54f) or ICY.

WES variants from patients were filtered using Ingenuity Variant Analysis, and verified using Integrative Genomics Viewer (IGV).
Gene knockout sequencing: Synthego Performance Analysis, ICE Analysis. Synthego (used in 2023)

For manuscripts utilizing custom algorithms or software that are central to the research but not yet described in published literature, software must be made available to editors and reviewers. We strongly encourage code deposition in a community repository (e.g. GitHub). See the Nature Portfolio guidelines for submitting code & software for further information.

Data

Policy information about availability of data

All manuscripts must include a data availability statement. This statement should provide the following information, where applicable:

- Accession codes, unique identifiers, or web links for publicly available datasets
- A description of any restrictions on data availability
- For clinical datasets or third party data, please ensure that the statement adheres to our policy

The following public datasets were used in the study: Gene Ontology annotations (http://download.baderlab.org/EM_Genesets/October_01_2020/Human/UniProt/Human_GO_AllPathways_with_GO_iea_October_01_2020_UniProt.gmt, v2020.10) and (http://download.baderlab.org/EM_Genesets/December_01_2021/Human/UniProt/Human_GO_AllPathways_with_GO_iea_December_01_2021_UniProt.gmt, v2021.12); IntAct Protein Interactions (https://www.ebi.ac.uk/intact/, v2019.12); IntAct Protein Complexes (https://www.ebi.ac.uk/complexportal/home, v2019.12); CORUM Protein Complexes (http://mips.helmholtz-muenchen.de/corum/download/allComplexes.xml.zip, v2019); Reactome Functional Interactions (https://reactome.org/download/tools/ReatomeFIs/FIsInGene_122220_with_annotations.txt.zip, v2020.12); BioGRID (https://downloads.thebiogrid.org/File/BioGRID/Release-Archive/BIOGRID-3.5.178/BIOGRID-ALL-3.5.178.psi25.zip, v2019.10), VirHostNet (v2.0; 2019.01); Human (versions 2018.02 and 2019.12), and VZV pOka (version 2017.12) protein sequences(https://uniprot.org); Human genomes GRCh38 (https://www.ncbi.nlm.nih.gov/assembly/GCF_000001405.26/) (transcriptome analysis) and GRCh37 (hg19, https://www.ncbi.nlm.nih.gov/assembly/GCF_000001405.13/) (WES). VZV genome from GenBank (https://www.ncbi.nlm.nih.gov/nuccore/NC_001348.1)

The mass spectrometry proteomics data have been deposited to the ProteomeXchange Consortium via the PRIDE partner repository with the following dataset identifiers: Full proteome of VZV-infected: PXD047273; VZV ORF interactomes: PXD047821; VZV ORF effectomes: PXD047362; Validation of knockouts: PXD047575; Full proteome of MPP8-knockout cells: PXD047393; NPHP4 interactomes: PXD061602.
The data and analysis results are accessible online via the interactive web interface at https://varizonet.innatelab.org.
The VZV ORF interactomes have been submitted to the IMEx (http://www.imexconsortium.org) consortium through IntAct and assigned the identifier IM-30341.

The transcriptome data (NPHP4-knockout SK-N-BE2 cells mock- and VZV-infected) have been deposited in the European Nucleotide Archive (ENA) at EMBL-EBI under accession number PRJEB86994.

Research involving human participants, their data, or biological material

Policy information about studies with human participants or human data. See also policy information about sex, gender (identity/presentation), and sexual orientation and race, ethnicity and racism.

Reporting on sex and gender	Sex and gender of the participants are reported in Supplementary Table S6-1, for information. However, none were analyzed nor used to claim any conclusion in this study.
Reporting on race, ethnicity, or other socially relevant groupings	N/A. Race, ethnicity, or other socially grouping of the participants were neither analyzed nor used to claim any conclusion in this study.
Population characteristics	The patient cohort consisted of adults (>18 years) with a final diagnosis of VZV-associated encephalitis or meningoencephalitis or cerebral vasculitis. A total of thirteen adult patients were included on the basis of pleocytosis and a positive PCR for VZV in the cerebrospinal fluid (CSF). PCR on CSF for HSV-1, HSV-2, and enterovirus as well as bacterial cultures were also performed and were negative for all patients included. Exclusion criteria were immunosuppressive therapy, known malignant disease, pregnancy, and HIV positivity. All patients had experienced chickenpox in childhood and none had received VZV vaccination.
Recruitment	The patients were recruited after their admission to the Department of Infectious Diseases at Aarhus University Hospital (AUH) with a final diagnosis of VZV-associated encephalitis or meningoencephalitis or cerebral vasculitis. All referred patients were admitted and included in the study if consent to participate was obtained. We can not exclude that the most severely ill patients were not included to to unconsciousness or a rapidly fatal disease course. There is no way to correct for this but we can not exclude to have missed patients with pathological variants and thus the estimated occurence/frequency of variants may be an underestimation.
Ethics oversight	The patients were included following oral and written consent in accordance with The Helsinki Declaration and national ethics guidelines and after approval from the Danish National Committee on Health Research Ethics (# 1-10-72-275-15), the Data Protection Agency, and Institutional Review Board.

Note that full information on the approval of the study protocol must also be provided in the manuscript.

Field-specific reporting

Please select the one below that is the best fit for your research. If you are not sure, read the appropriate sections before making your selection.

☒ Life sciences ☐ Behavioural & social sciences ☐ Ecological, evolutionary & environmental sciences

For a reference copy of the document with all sections, see nature.com/documents/nr-reporting-summary-flat.pdf

Life sciences study design

All studies must disclose on these points even when the disclosure is negative.

Sample size	The sample sizes were chosen from past knowledge on the good sample size to ensure adequate power and reproducibility (Blainey et al., 2014 https://doi.org/10.1038/nmeth.3091; Conesa et al., 2016 https://doi.org/10.1186/s13059-016-0881-8). Sample sizes are always indicated in figure legends or related "Methods" section.
Data exclusions	MS runs of the effectome datasets which were not reaching satisfying performance were excluded. This led to the removal of the data for 18 VZV ORFs.
Replication	For mass spectrometry, in vitro viral replication validation experiments and cell viability assays, a minimum of three biological experiments were performed independently.<br>Validation of the VZV ORF32 and GTF2B interaction by co-IP was performed from two independent biological experiments, each based on independent samples analysed with a given approach (viral protein IP and reversed host protein IP).<br>Immunofluorescence analysis of VZV ORF32 and GTF2B localisation was performed on 20 cells, across two independent biological experiments. Immunofluorescence-based quantification of IFI16 expression in infected cells was performed on at least 120 cells per conditions, across three independent biological experiments. Immunofluorescence-based quantification of IFI16 expression in ORF61-expressing cells was performed on at least 500 cells per conditions, across two independent biological experiments. Immunofluorescence analysis of VZV ORF61 and UBXN7 localisation was performed on 20 cells, across three independent biological experiments.<br>All attempts at replication of the co-IP and immunofluorescence experiments were successful.<br>The host gene knockout screen on viral replication was conducted with two technical replicates per condition per sgRNA, with three to four sgRNA reagents used independently per tested gene.<br>Knockout of genes of interest were reproduced using the same sgRNA as used in the screen, in independent cell batches, and validated in two to three independent samples each analyzed by a given technique (genome sequencing, western-blot, mass spectrometry).
Randomization	Samples for AP-MS experiments were randomzied to avoid carry-over bias during the MS analysis. No randomization was used otherwise given the small number of samples and the lack of influence of randomization on the experimental design and experimental approach used. (no animal experiments were performed in this study).
Blinding	N/A. Investigators were not blinded to experimental groups (in vitro experiments required prior knowledge for data interpretation)

Reporting for specific materials, systems and methods

We require information from authors about some types of materials, experimental systems and methods used in many studies. Here, indicate whether each material, system or method listed is relevant to your study. If you are not sure if a list item applies to your research, read the appropriate section before selecting a response.

Materials & experimental systems

n/a	Involved in the study	
☐	☒	Antibodies
☐	☒	Eukaryotic cell lines
☒	☐	Palaeontology and archaeology
☒	☐	Animals and other organisms
☒	☐	Clinical data
☒	☐	Dual use research of concern
☒	☐	Plants

Methods

n/a	Involved in the study	
☒	☐	ChIP-seq
☐	☒	Flow cytometry
☒	☐	MRI-based neuroimaging

Antibodies

Antibodies used	Primary antibodies used in this study were the following: TFIIB (Cell Signaling Technology, 4169; WB 1:1000), MPP8 (Proteintech, 16796; WB 1:1500), ZNF280D (Invitrogen, PA5-56410; WB 1:1000), NPHP4 (Abclonal, A8934; WB 1:3000), UBXN7 (Sigma-Aldrich, HPA049442; IF 1:500), IFI16 (Cell Signaling Technology, 14970; WB 1:1000; IF 1:500), V5-tag rabbit (Cell Signaling Technology, 13202; WB 1:1000; IF 1:1000), V5-tag mouse (Invitrogen, R960-25; WB 1:1000; IF 1:400), HA-tag (Cell Signaling Technology, 2367; WB 1:2500; IF 1:100), HA-tag-HRP (Sigma-Aldrich, H6533; WB 1:1000), β-actin-HRP (Santa Cruz, sc-47778; WB 1:2500), β-tubulin (Cell Signaling Technology, 2128; WB 1:500). For western-blot, secondary antibodies conjugated to HRP detecting rabbit IgG (1:2500) and mouse IgG (1:5000) were purchased from Dako and Sigma-Aldrich, respectively. For immunofluorescence, DAPI (1:1000) and secondary antibody detecting rabbit or mouse IgG conjugated to Alexa 488, 594 or 647 (1:200-1:500) were purchased from Invitrogen. GFP-DyLight-488 was purchased from Rockland (600-141-215; 1:1000).
Validation	The supplier of the TFIIB antibody claims for specificity for Human, Mouse, Rat, Monkey species ("TFIIB (2F6A3H4) Mouse mAb detects endogenous levels of total TFIIB protein.") and provides western blot data for the evaluation of its specificity in human cells.<br>The specificity of the MPP8 antibody was validated for western blot using control and knockdown human cells in this study https://doi.org/10.1038/s41564-018-0256-x, Figure 2c.<br>The supplier of the NPHP4 antibody provides western blot data for the evaluation of its specificity in human cells.

The supplier of the UBXN7 provides immunofluorescence data for the evaluation of its specificity in human cells. "enhanced" validations for immunofluorescence are available at https://www.proteinatlas.org/ENSG00000163960-UBXN7/summary/antibody (using human cells with variable expression levels and cross-validation with other antibodies).
The supplier of the ZNF280D antibody provides data for the evaluation of its specificity in human cells. The datasheet claims that it was tested for western blot.
The supplier of the IFI16 antibody provides western blot data for the evaluation of its specificity in human cells. The specificity was also validated for western blot using control and knockdown human cells in this study https://doi.org/10.1016/j.chom.2018.01.012, Figure 5a.
The suppliers of the V5-tag (rabbit and mouse) and the HA-tag antibodies provide western blot and immunofluorescence data for the evaluation of their specificity.
The suppliers of the HA-tag-HRP and β-actin-HRP antibodies provide western blot data for the evaluation of their specificity.
The supplier of the β-tubulin antibody provides references showing its specificity in human cells for western blot analysis.
The supplier of the GFP-DyLight-488 antibody provides immunofluorescence data showing its specifity.

Eukaryotic cell lines

Policy information about cell lines and Sex and Gender in Research

Cell line source(s)

SK-N-BE2 cells (CRL-2271) were kindly provided by Rüdiger Klein (MPI of Neurobiology, Munich, Germany). MeWo cells (HTB-65TM) were kindly provided by Abel-Viejo Borbolla (MHH, Hannover, Germany). HEK293T (CRL-11268) were purchased from ATCC. HeLa Kyoto expressing GFP-tagged GTF2B from BAC transgene was from Ina Poser. HFF-1 (SCRC-1041) cells were a kind gift from Prof. Melanie Brinkmann (HZI, Braunschweig, Germany).

Authentication

Authentication was performed by STR profiling.

Mycoplasma contamination

All cell lines were tested to be mycoplasma free by standard PCR-based assay.

Commonly misidentified lines
(See ICLAC register)

No commonly misidentified cell lines were used in this study.

Plants

Seed stocks

N/A

Novel plant genotypes

N/A

Authentication

N/A

Flow Cytometry

Plots

Confirm that:

- [x] The axis labels state the marker and fluorochrome used (e.g. CD4-FITC).
- [x] The axis scales are clearly visible. Include numbers along axes only for bottom left plot of group (a 'group' is an analysis of identical markers).
- [x] All plots are contour plots with outliers or pseudocolor plots.
- [x] A numerical value for number of cells or percentage (with statistics) is provided.

Methodology

Sample preparation

FACS analysis of VZV rOka infection: SK-N-BE2 cells infected with VZV rOka were fixed with 3.7% Formaldehyde (Sigma-Aldrich) for 15 minutes at room temperature. After a wash in FACS buffer (1% FCS, 2mM EDTA in PBS), cells were labelled with the Varicella Zoster (VZV) DFA Kit reagent (Merck, Light Diagnostic), containing two FITC conjugated antibodies against VZV immediate-early ORF62 protein and late glycoprotein E for 15 minutes at room temperature.
FACS analysis of VZV-RFP infection: SK-N-BE2 cells knockout for the gene of interest or controls and infected with the VZV-RFP reporter virus were fixed in 1% PFA for 15 minutes and resuspended in FACS buffer (5mM EDTA pH8, 25mM Hepes, 1% FCS in PBS).

Instrument

VZV rOka infected samples were analyzed on an Attune NxT Acoustic Focusing Cytometer (Thermo Fisher Scientific).
Reporter VZV-RFP-infected samples were analyzed on a cytoFLEX (Beckman Coulter).

Software	Data were acquired using the software provided with the given instrument by the supplier. FlowJo 10 was used for data analysis.
Cell population abundance	Full proteome analysis if VZV-infected cells: living cells represented (median value) 61% of the total acquired objects. Single cells represented (median value) 70% of the parent living cell population. FITC threshold was set from mock samples at 2000. FITC negative cells represented (median value) 99.8% of the single cell population in mock samples.<br>KO screen: living cells represented (median value) 74% of the total acquired objects. Single cells represented (median value) 71% of the parent living cell population. Co-cultured GFP and BFP cells represented (median value) 44% and 38%, respectively, of the parent single cell population, in mock wells.
Gating strategy	Forward and side scatters were used to 1) gate living cell and exclude cell debris (SSC-A/FSC-A), 2) gate single cells (FSC-H/FSC-A).<br>VZV rOka infected cells were gated from single cells as FITC positive.<br>In the reporter VZV-RFP replication screen, co-cultured knockout and control cells were gated from single cells as BFP and GFP positive cells, respectively, in order to excluded unmarked inoculum cells. The median RFP intensity of each population was used to assess VZV propagation. For follow-up validations performed in knockout cells only, knockout cells were isolated from the inoculum cells, within the single cells population, as BFP positive cells.

☒ Tick this box to confirm that a figure exemplifying the gating strategy is provided in the Supplementary Information.
