# Supplementary figures and images for: Multi-proteomic profiling of the varicella-zoster virus–host interface reveals host susceptibilities to severe infection
Source: Nat Microbiol. 2025 Jul 30;10(8):2048–72. doi: 10.1038/s41564-025-02068-7 (PMC12313529; doi:10.1038/s41564-025-02068-7)

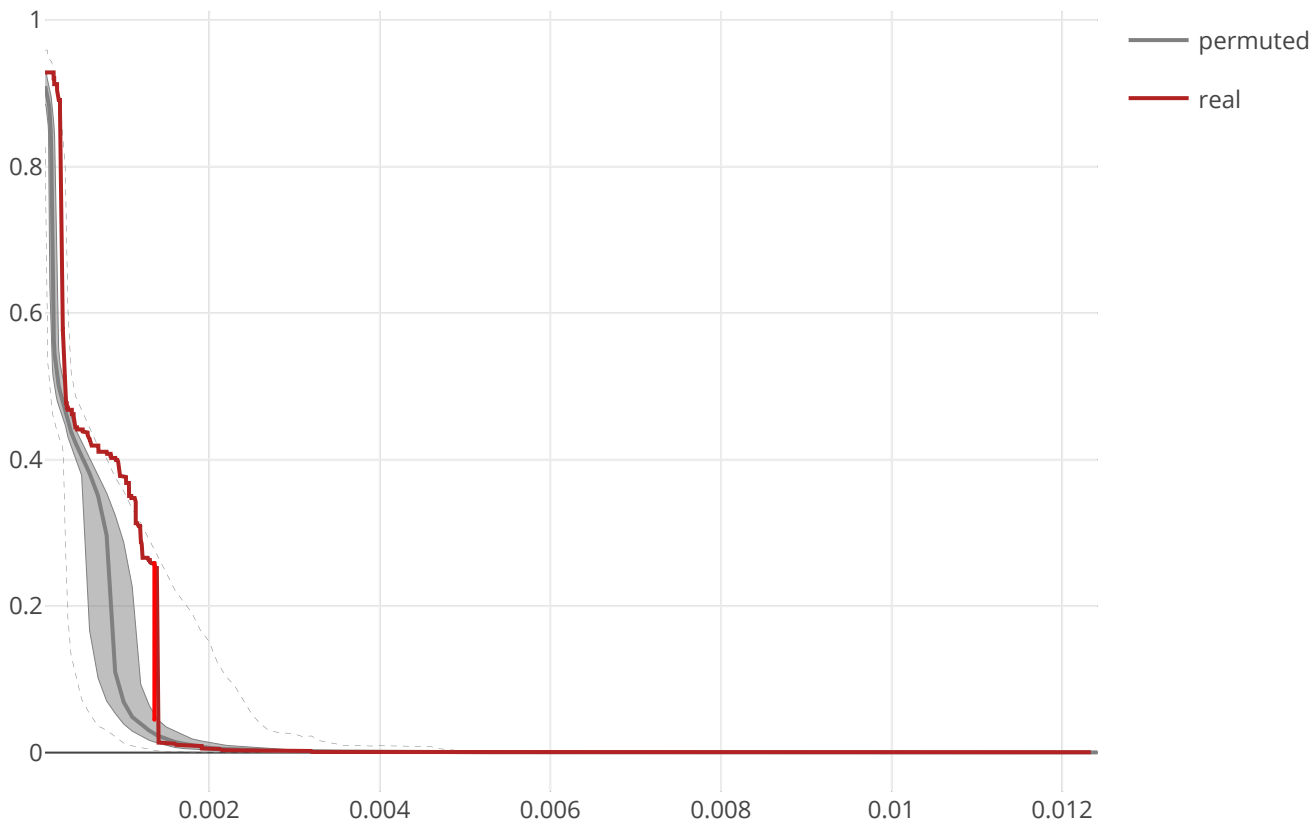

Supplement: Supplementary file 12 — VZV ORF subnetworks integrating interactome and effectome data via the HotNet-based network diffusion method and plots of the distribution of their average inverted length of target-effect paths across HotNet threshold resulting from real data compared with random permuted data. [file 41564_2025_2068_MOESM12_ESM.zip › metrics/VZV-10_flow_avginvlen.pdf]

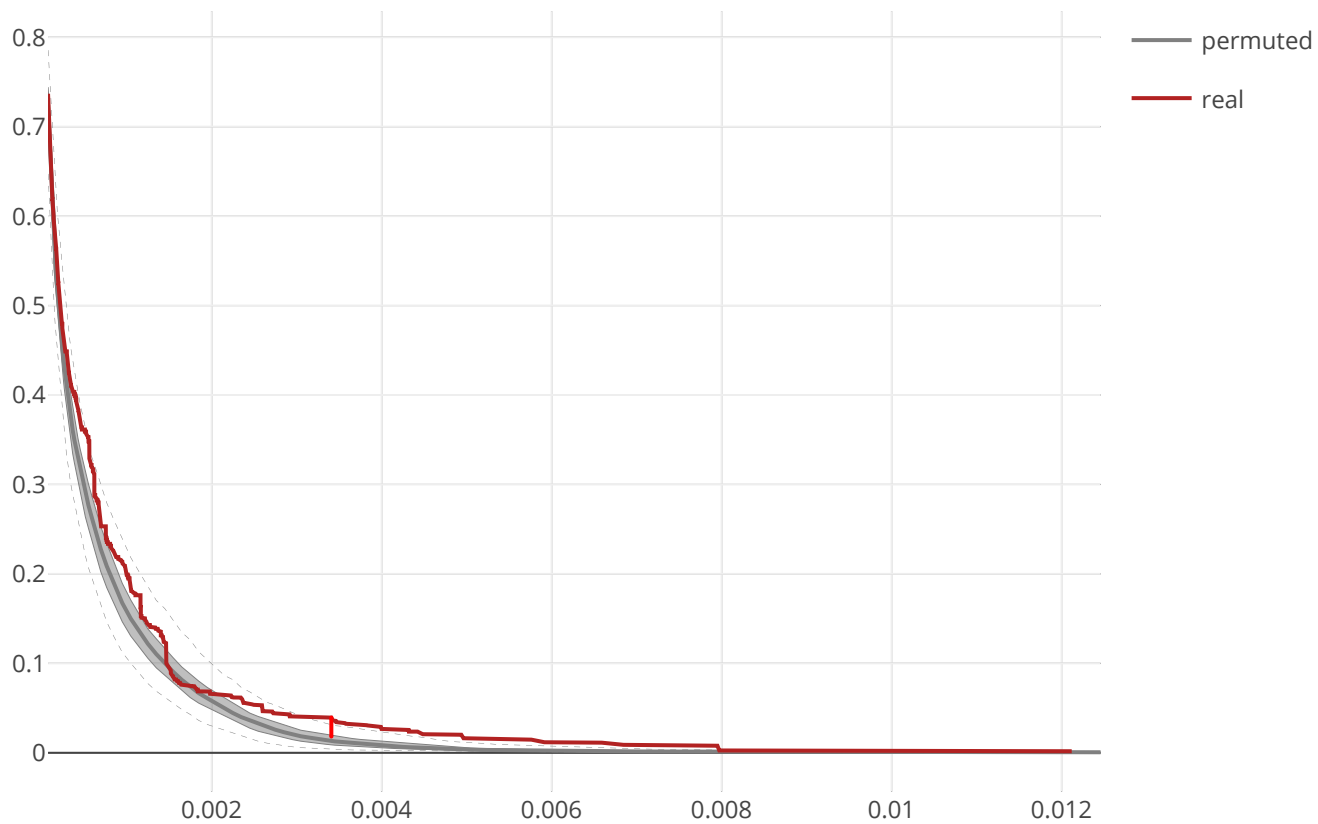

Supplement: Supplementary file 12 — VZV ORF subnetworks integrating interactome and effectome data via the HotNet-based network diffusion method and plots of the distribution of their average inverted length of target-effect paths across HotNet threshold resulting from real data compared with random permuted data. [file 41564_2025_2068_MOESM12_ESM.zip › metrics/VZV-12_flow_avginvlen.pdf]

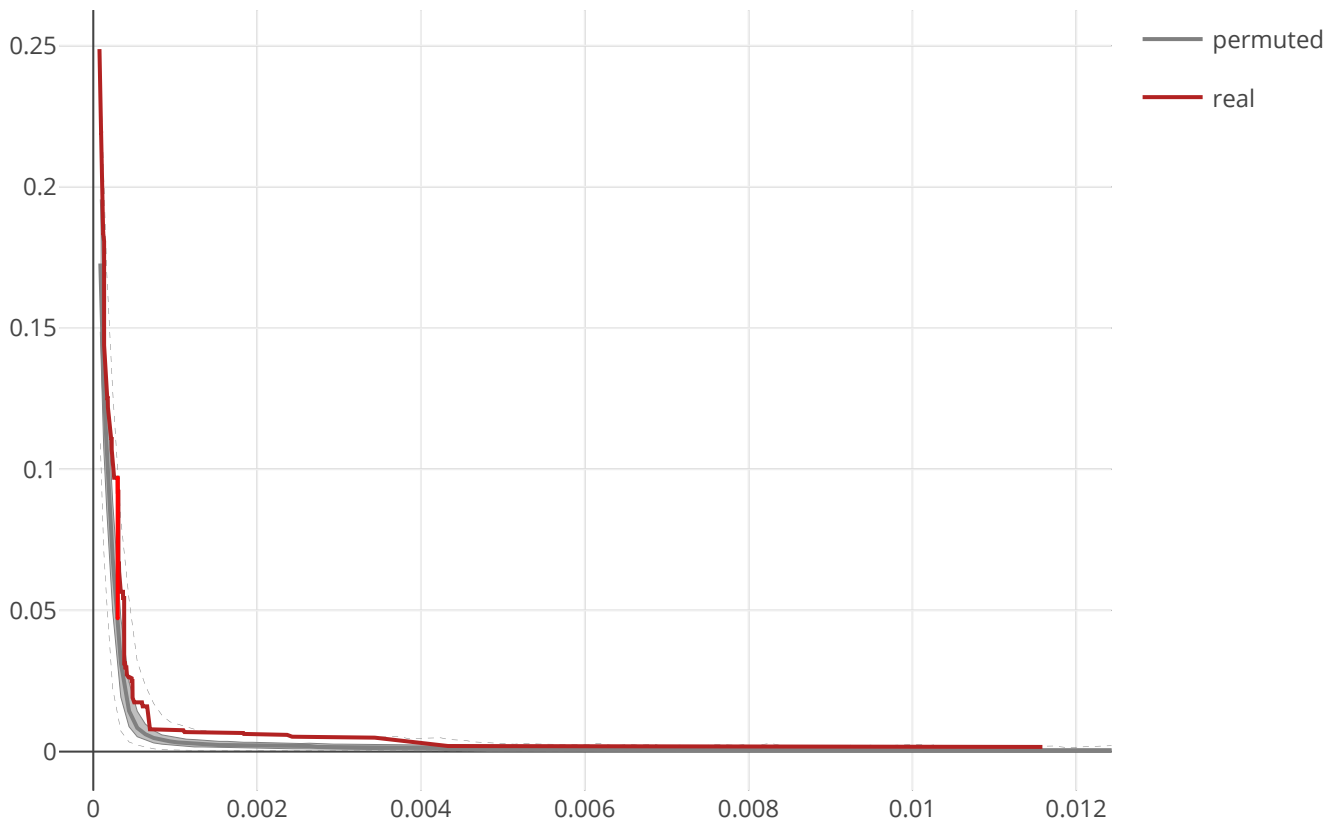

Supplement: Supplementary file 12 — VZV ORF subnetworks integrating interactome and effectome data via the HotNet-based network diffusion method and plots of the distribution of their average inverted length of target-effect paths across HotNet threshold resulting from real data compared with random permuted data. [file 41564_2025_2068_MOESM12_ESM.zip › metrics/VZV-49_flow_avginvlen.pdf]

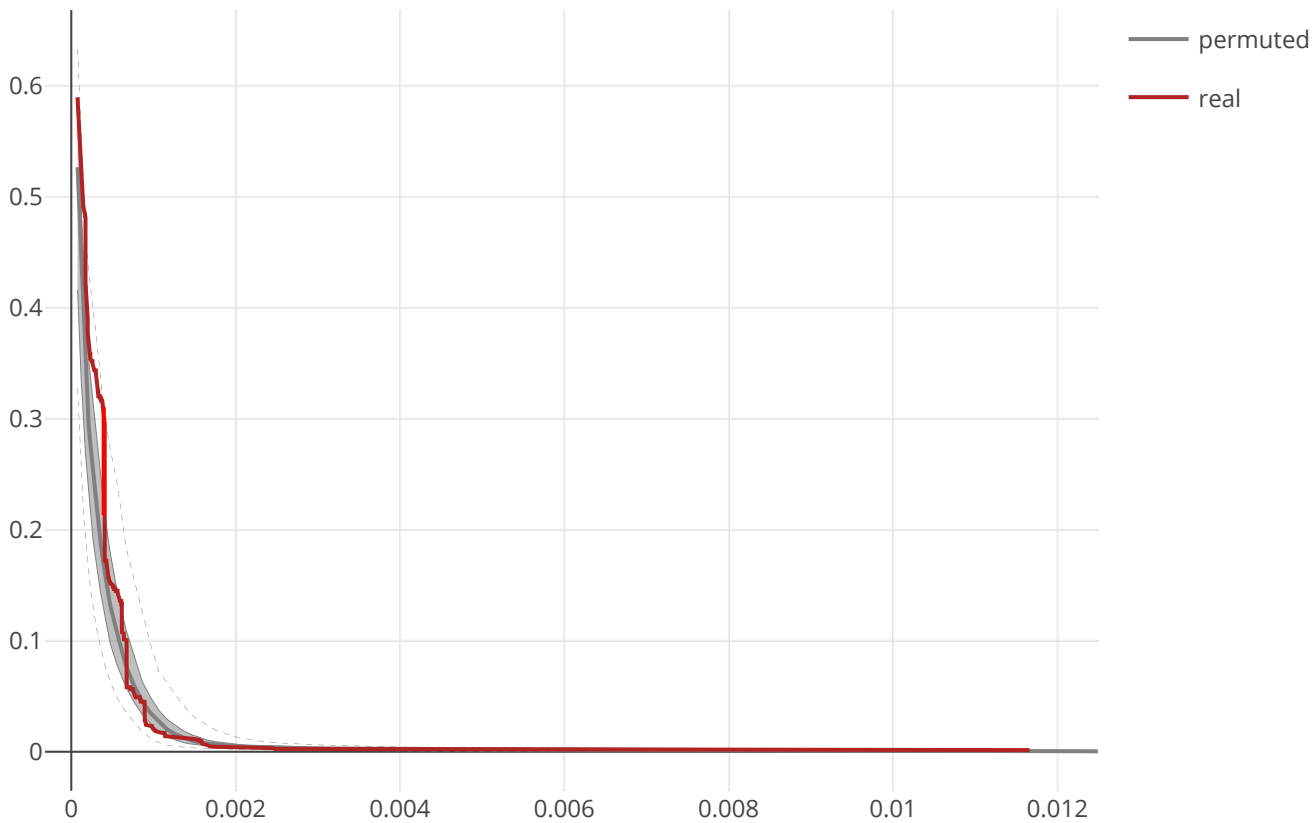

Supplement: Supplementary file 12 — VZV ORF subnetworks integrating interactome and effectome data via the HotNet-based network diffusion method and plots of the distribution of their average inverted length of target-effect paths across HotNet threshold resulting from real data compared with random permuted data. [file 41564_2025_2068_MOESM12_ESM.zip › metrics/VZV-4_flow_avginvlen.pdf]

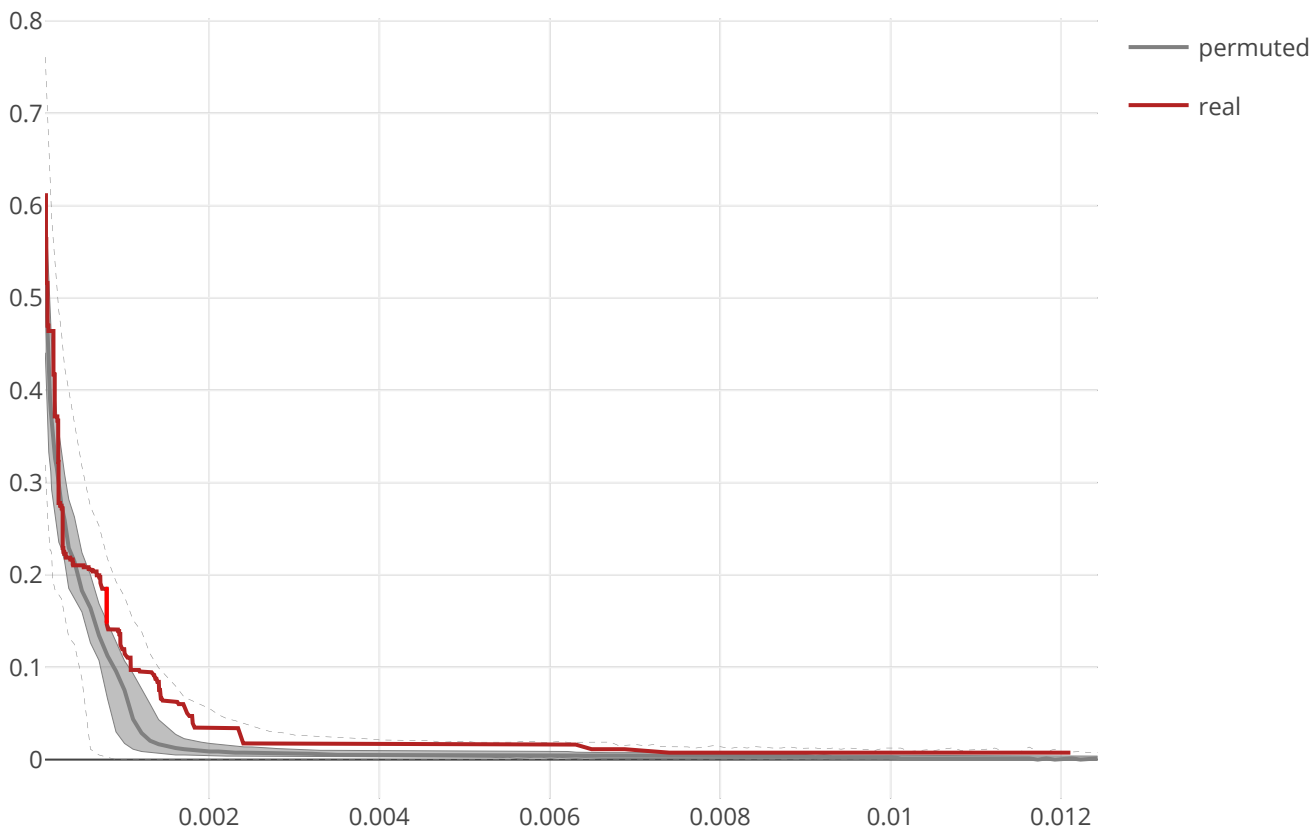

Supplement: Supplementary file 12 — VZV ORF subnetworks integrating interactome and effectome data via the HotNet-based network diffusion method and plots of the distribution of their average inverted length of target-effect paths across HotNet threshold resulting from real data compared with random permuted data. [file 41564_2025_2068_MOESM12_ESM.zip › metrics/VZV-61_flow_avginvlen.pdf]

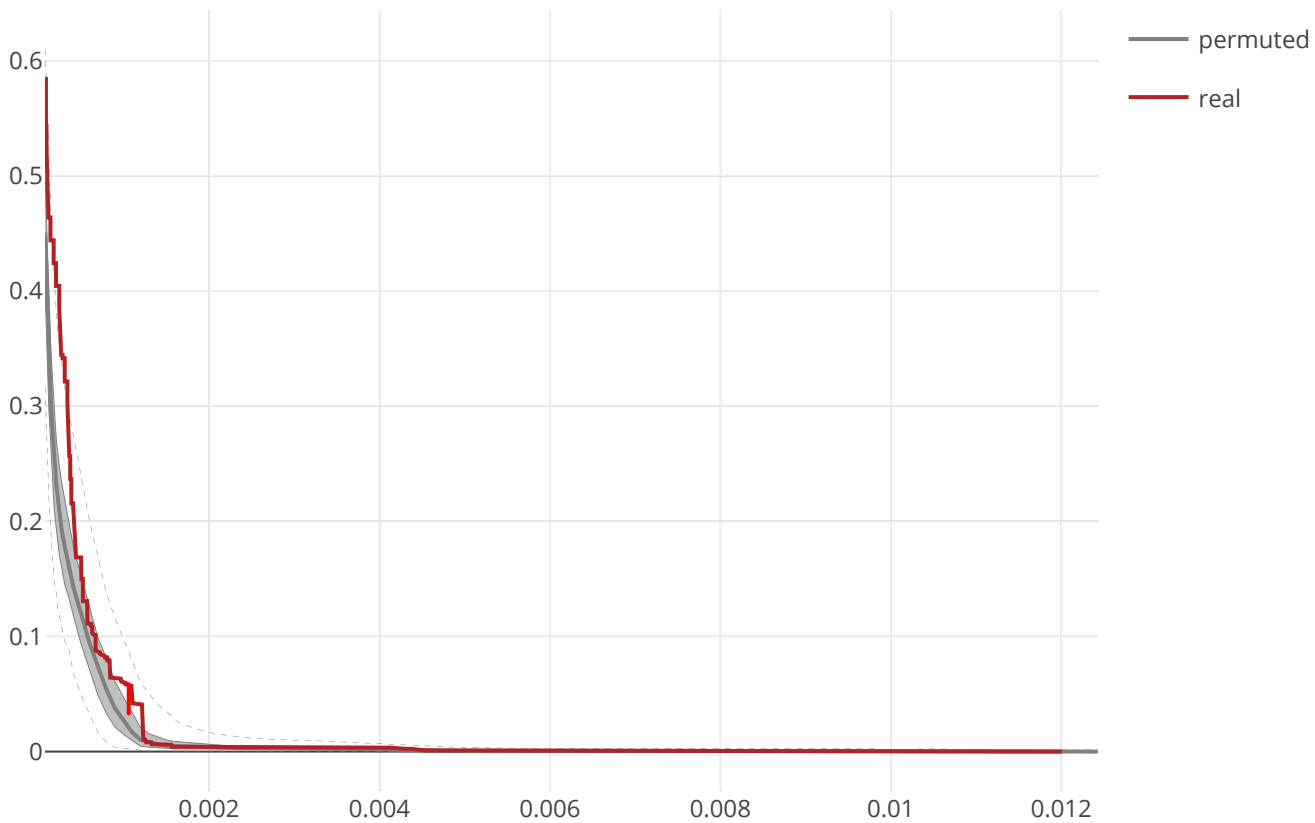

Supplement: Supplementary file 12 — VZV ORF subnetworks integrating interactome and effectome data via the HotNet-based network diffusion method and plots of the distribution of their average inverted length of target-effect paths across HotNet threshold resulting from real data compared with random permuted data. [file 41564_2025_2068_MOESM12_ESM.zip › metrics/VZV-66.2_flow_avginvlen.pdf]

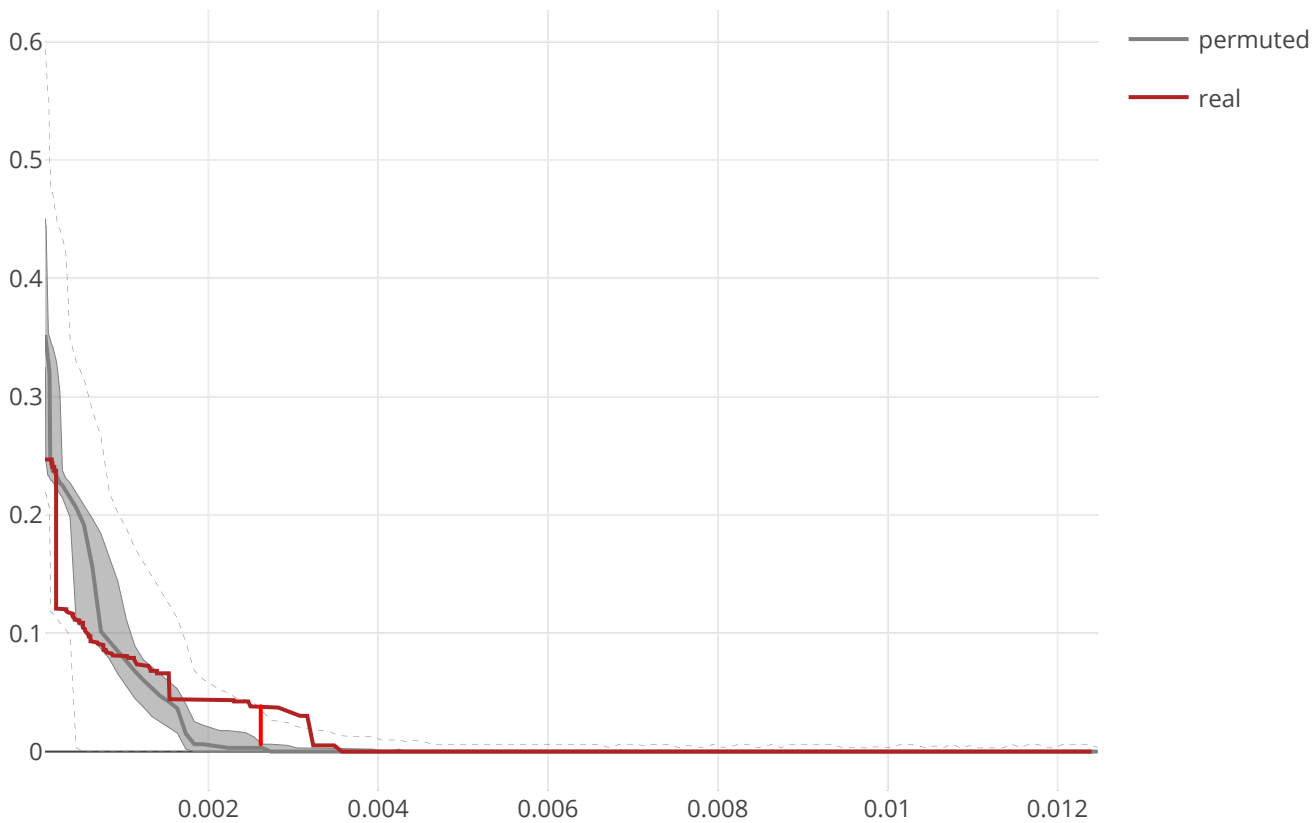

Supplement: Supplementary file 12 — VZV ORF subnetworks integrating interactome and effectome data via the HotNet-based network diffusion method and plots of the distribution of their average inverted length of target-effect paths across HotNet threshold resulting from real data compared with random permuted data. [file 41564_2025_2068_MOESM12_ESM.zip › metrics/VZV-7_flow_avginvlen.pdf]

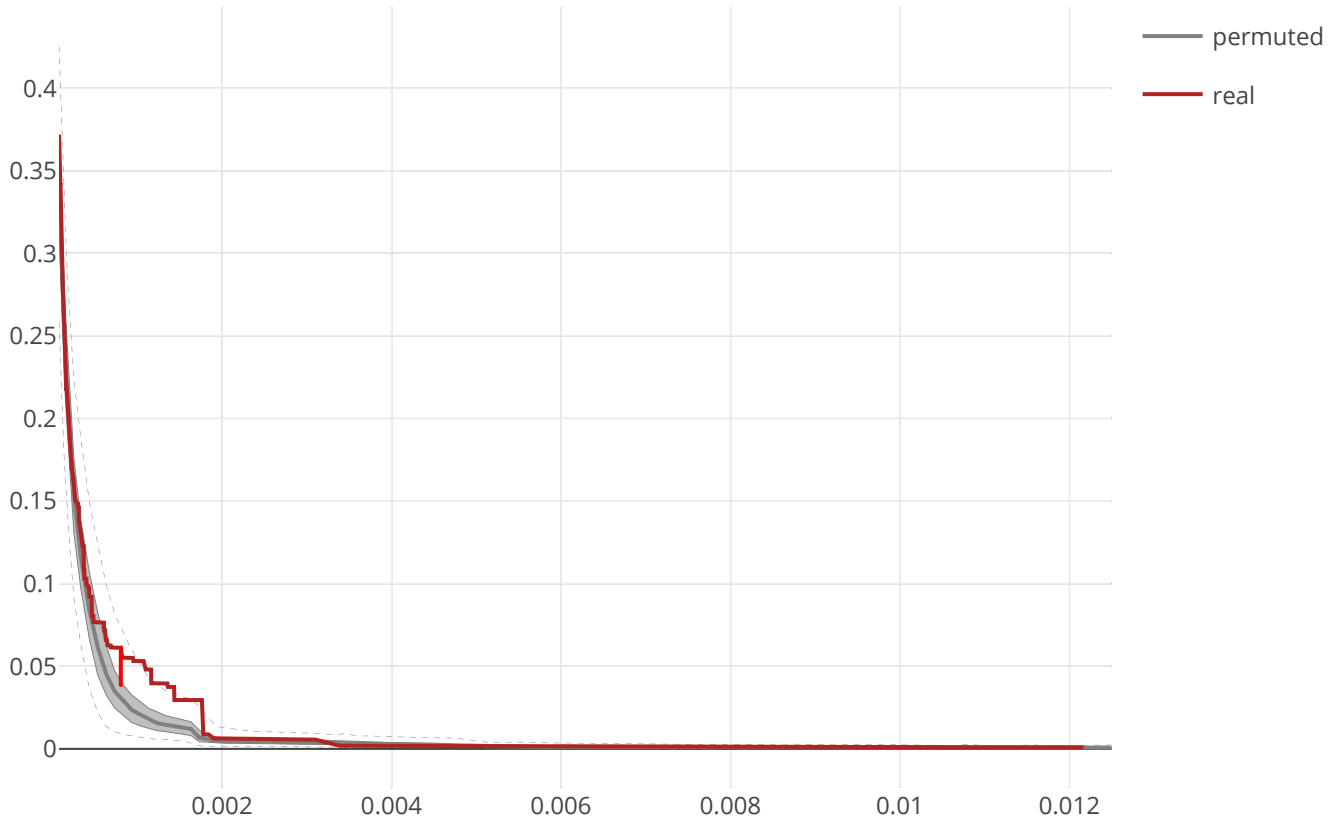

Supplement: Supplementary file 12 — VZV ORF subnetworks integrating interactome and effectome data via the HotNet-based network diffusion method and plots of the distribution of their average inverted length of target-effect paths across HotNet threshold resulting from real data compared with random permuted data. [file 41564_2025_2068_MOESM12_ESM.zip › metrics/VZV-9_flow_avginvlen.pdf]

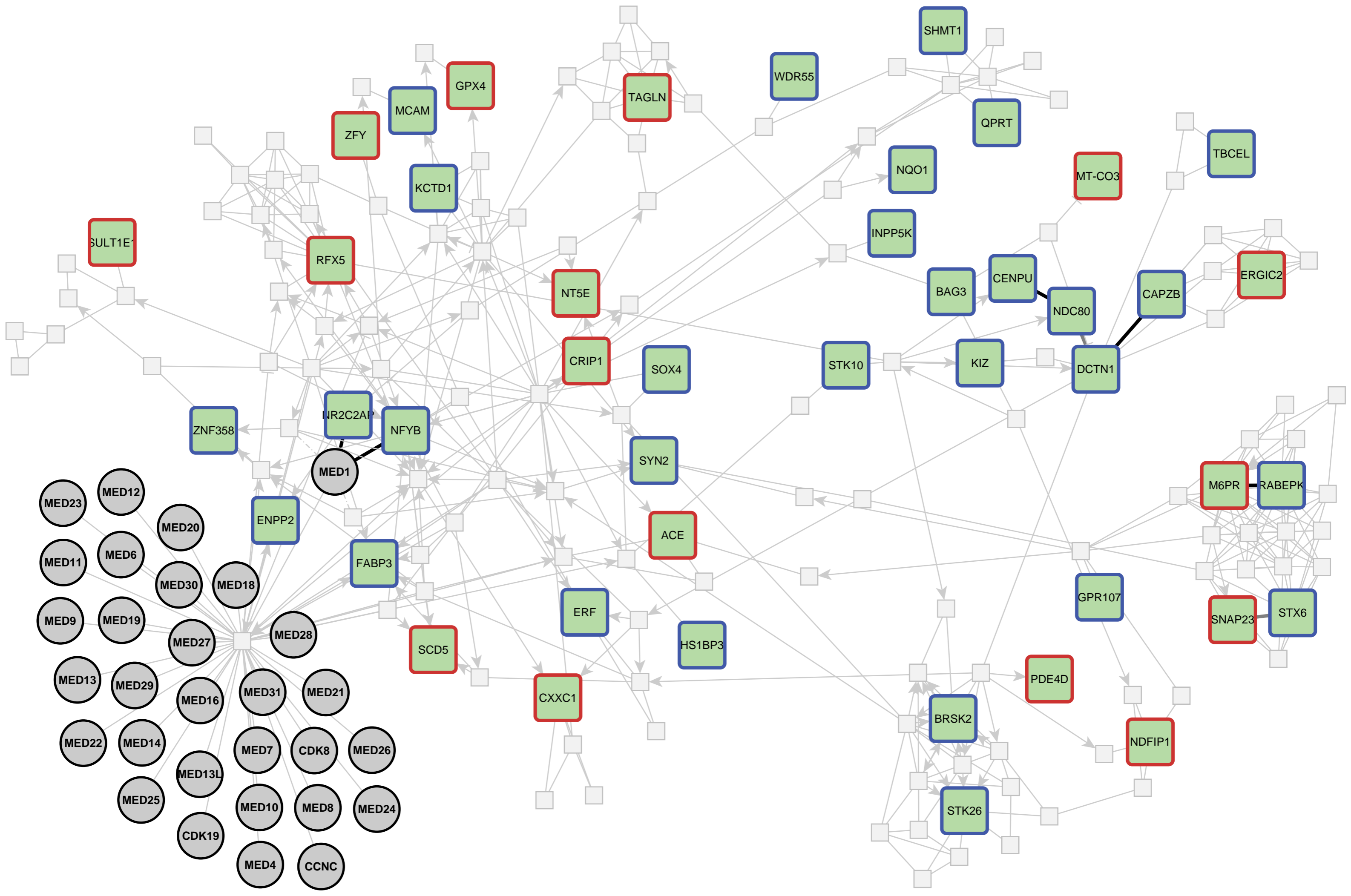

Supplement: Supplementary file 12 — VZV ORF subnetworks integrating interactome and effectome data via the HotNet-based network diffusion method and plots of the distribution of their average inverted length of target-effect paths across HotNet threshold resulting from real data compared with random permuted data. [file 41564_2025_2068_MOESM12_ESM.zip › networks_pdf/vgirault_vzvapms_VZV-10_flow_20210707_opt_adj_fig.pdf]

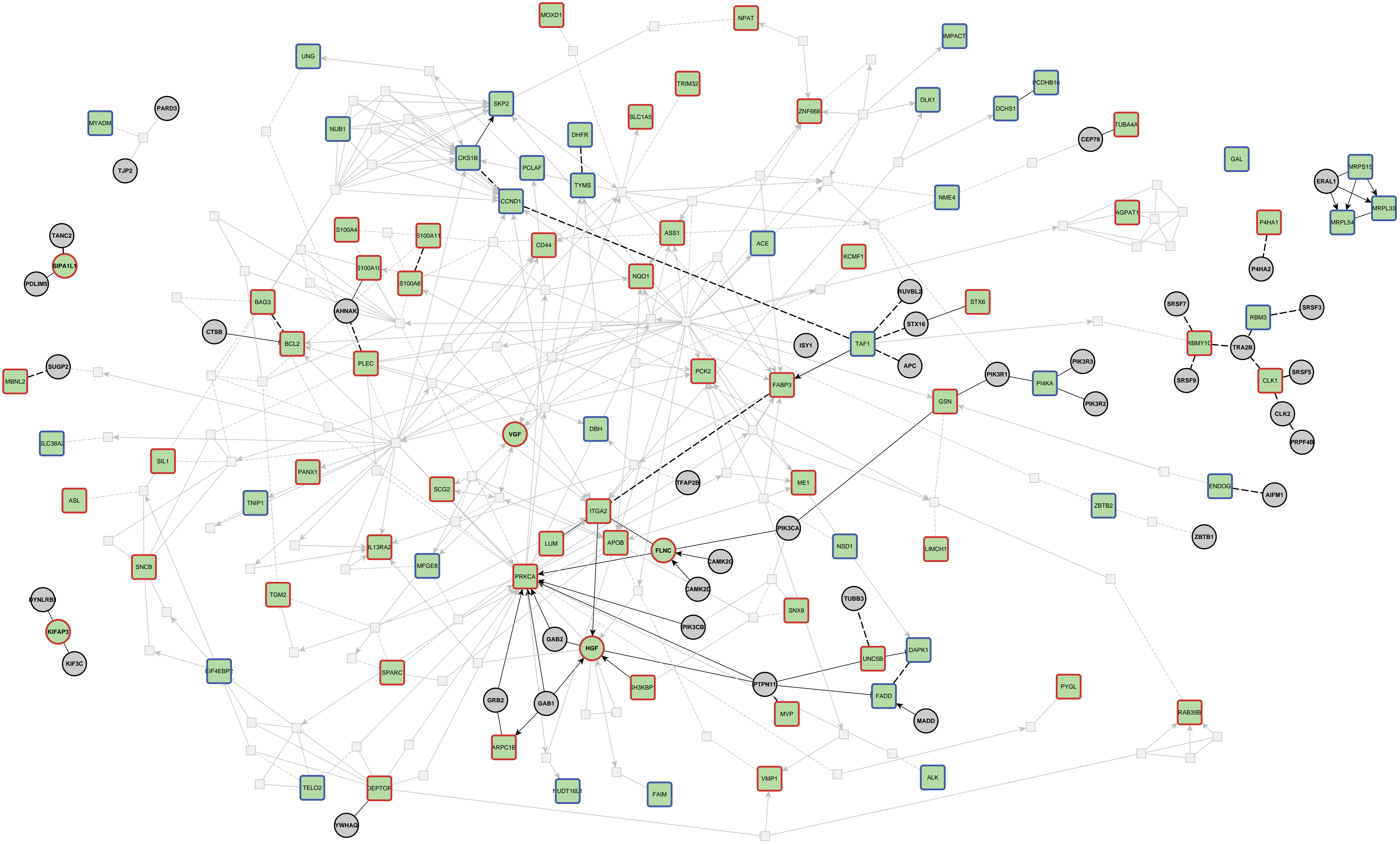

Supplement: Supplementary file 12 — VZV ORF subnetworks integrating interactome and effectome data via the HotNet-based network diffusion method and plots of the distribution of their average inverted length of target-effect paths across HotNet threshold resulting from real data compared with random permuted data. [file 41564_2025_2068_MOESM12_ESM.zip › networks_pdf/vgirault_vzvapms_VZV-12_flow_20210707_opt_adj_fig.pdf]

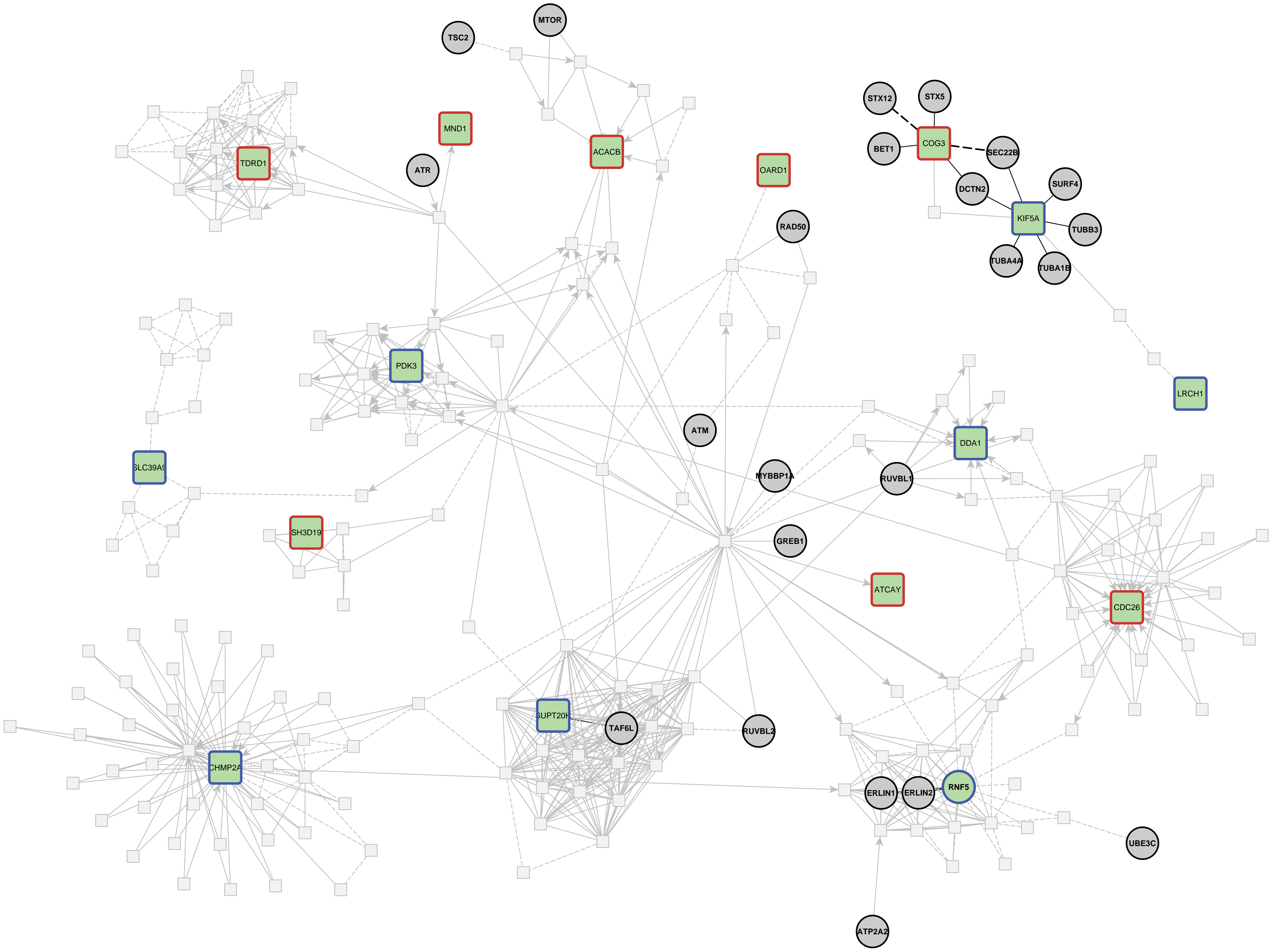

Supplement: Supplementary file 12 — VZV ORF subnetworks integrating interactome and effectome data via the HotNet-based network diffusion method and plots of the distribution of their average inverted length of target-effect paths across HotNet threshold resulting from real data compared with random permuted data. [file 41564_2025_2068_MOESM12_ESM.zip › networks_pdf/vgirault_vzvapms_VZV-49_flow_20210707_opt_adj_fig.pdf]

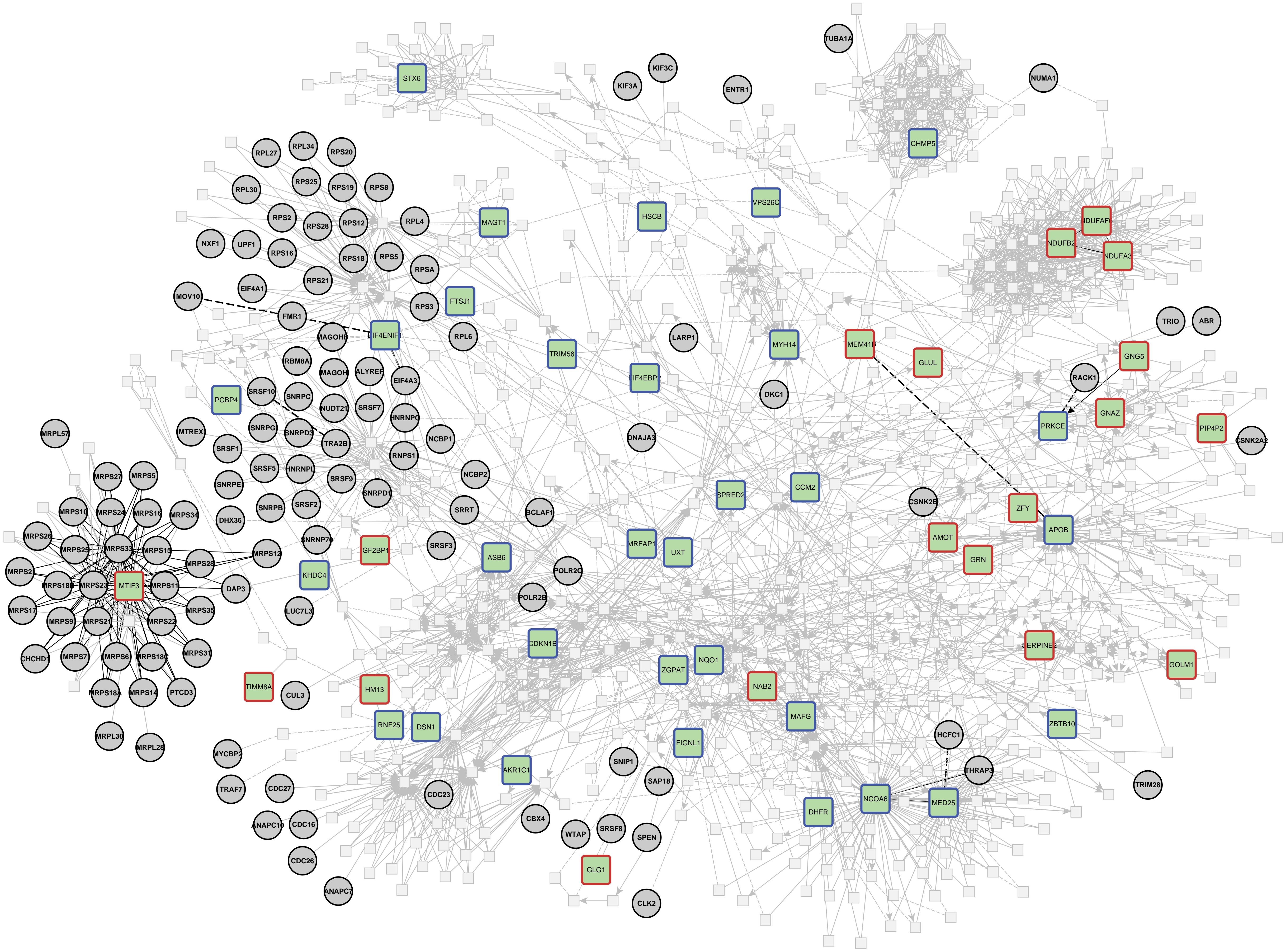

Supplement: Supplementary file 12 — VZV ORF subnetworks integrating interactome and effectome data via the HotNet-based network diffusion method and plots of the distribution of their average inverted length of target-effect paths across HotNet threshold resulting from real data compared with random permuted data. [file 41564_2025_2068_MOESM12_ESM.zip › networks_pdf/vgirault_vzvapms_VZV-4_flow_20210707_opt_adj_fig.pdf]

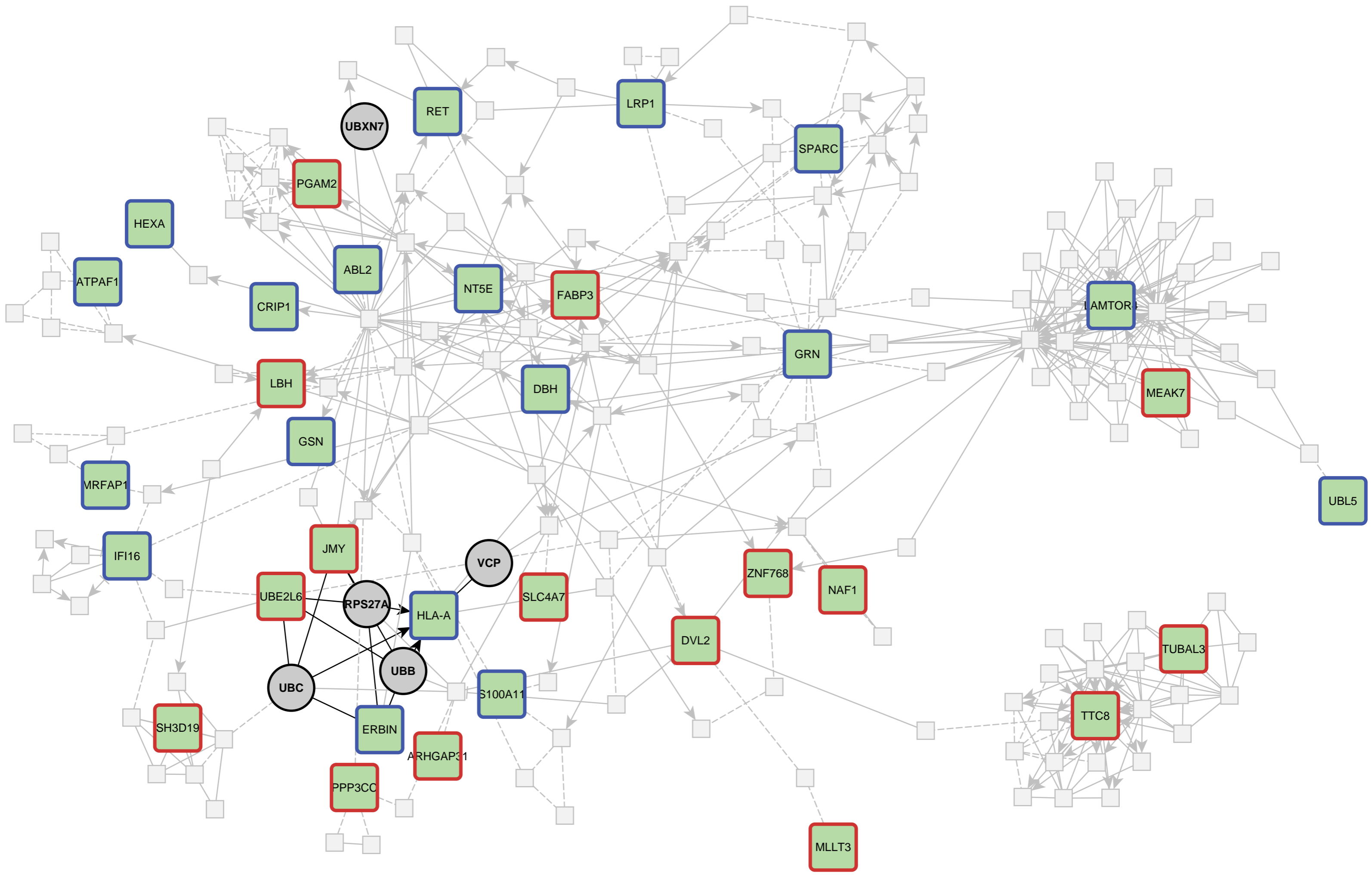

Supplement: Supplementary file 12 — VZV ORF subnetworks integrating interactome and effectome data via the HotNet-based network diffusion method and plots of the distribution of their average inverted length of target-effect paths across HotNet threshold resulting from real data compared with random permuted data. [file 41564_2025_2068_MOESM12_ESM.zip › networks_pdf/vgirault_vzvapms_VZV-61_flow_20210707_opt_adj_fig.pdf]

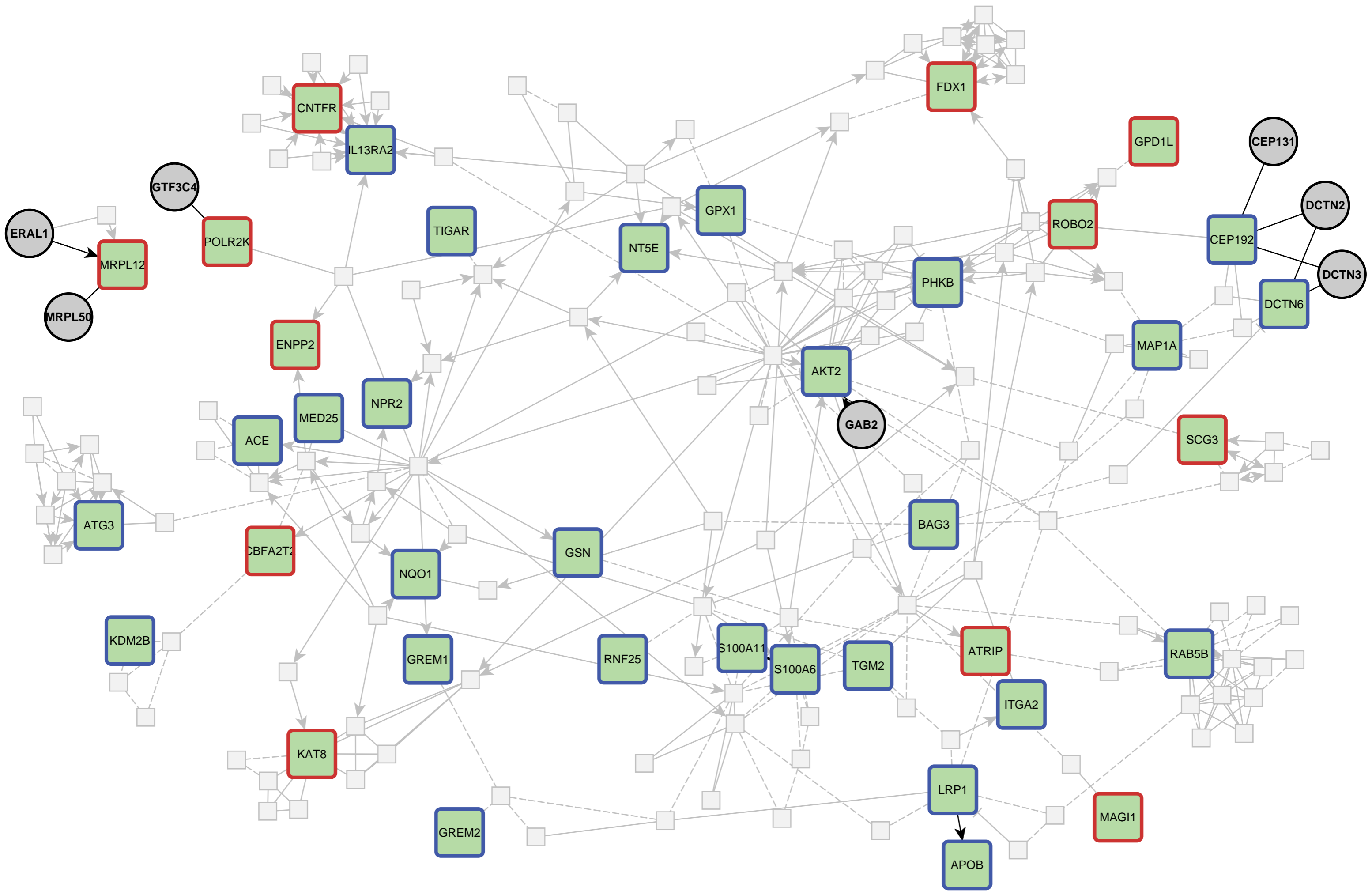

Supplement: Supplementary file 12 — VZV ORF subnetworks integrating interactome and effectome data via the HotNet-based network diffusion method and plots of the distribution of their average inverted length of target-effect paths across HotNet threshold resulting from real data compared with random permuted data. [file 41564_2025_2068_MOESM12_ESM.zip › networks_pdf/vgirault_vzvapms_VZV-66.2_flow_20210707_opt_adj_fig.pdf]

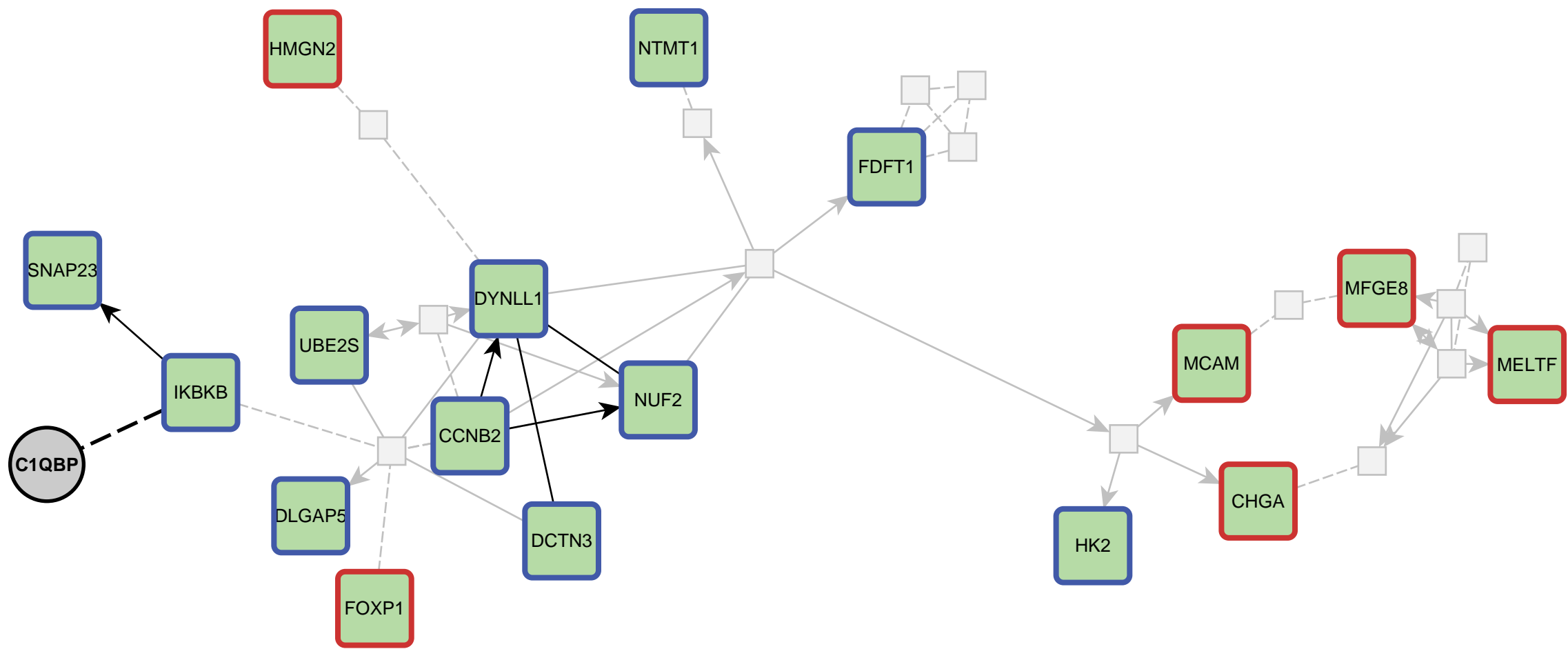

Supplement: Supplementary file 12 — VZV ORF subnetworks integrating interactome and effectome data via the HotNet-based network diffusion method and plots of the distribution of their average inverted length of target-effect paths across HotNet threshold resulting from real data compared with random permuted data. [file 41564_2025_2068_MOESM12_ESM.zip › networks_pdf/vgirault_vzvapms_VZV-7_flow_20210707_opt_adj_fig.pdf]

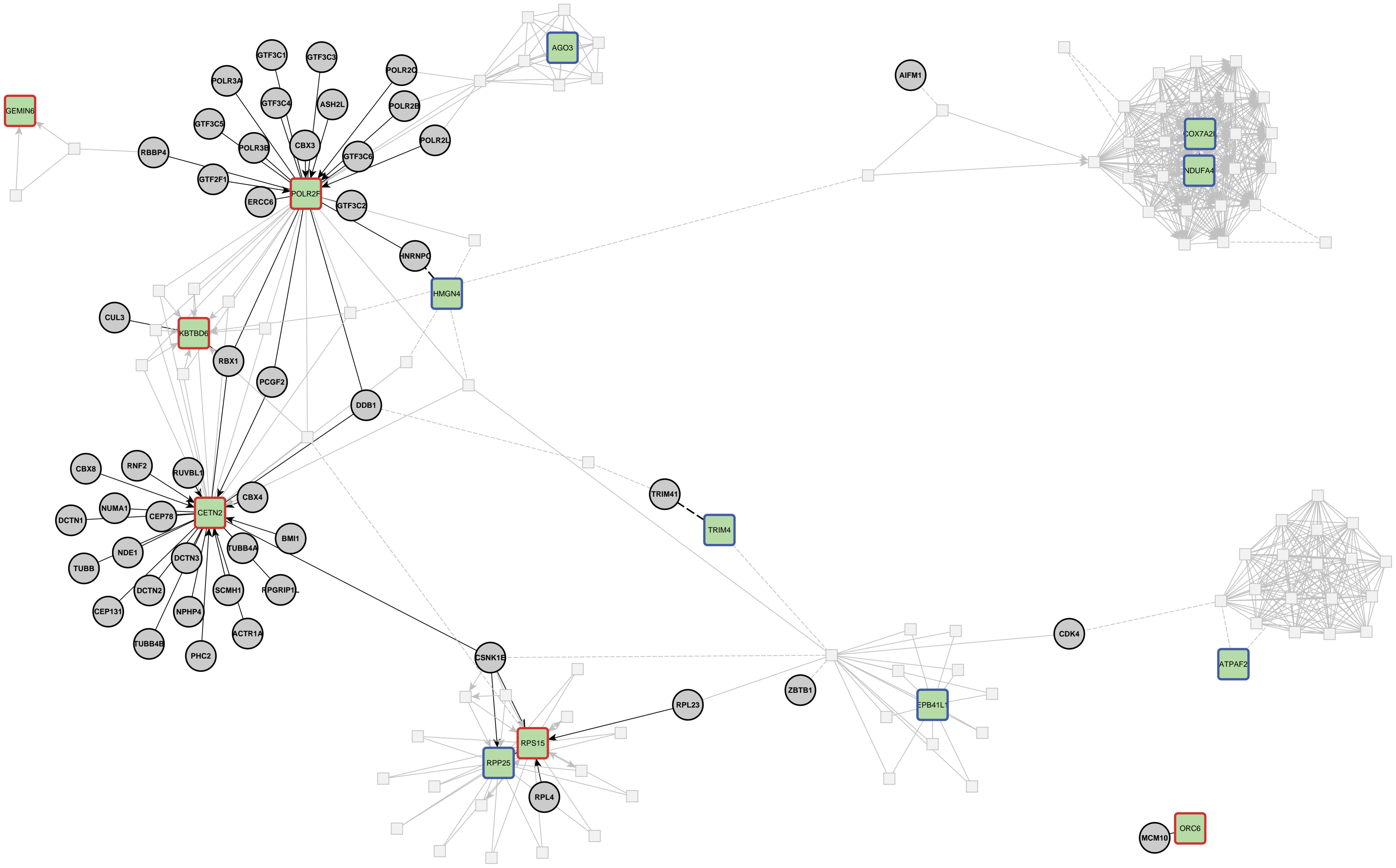

Supplement: Supplementary file 12 — VZV ORF subnetworks integrating interactome and effectome data via the HotNet-based network diffusion method and plots of the distribution of their average inverted length of target-effect paths across HotNet threshold resulting from real data compared with random permuted data. [file 41564_2025_2068_MOESM12_ESM.zip › networks_pdf/vgirault_vzvapms_VZV-9_flow_20210707_opt_adj_fig.pdf]

# Source Data 2 - Figure 4

Uncropped blots - Figure 4b

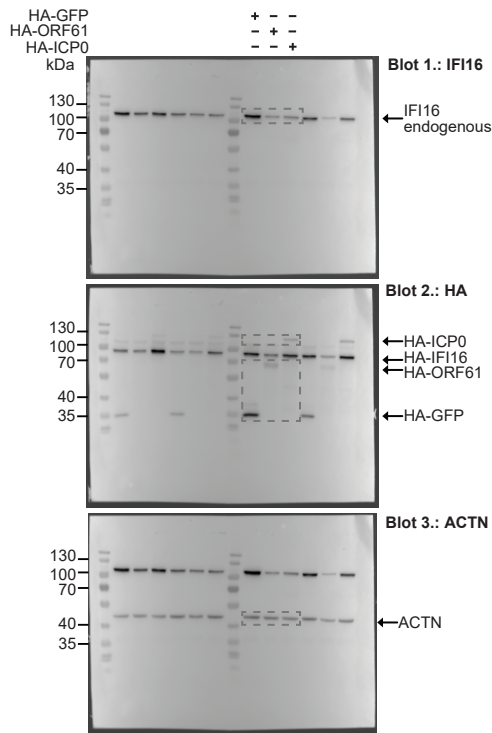

Supplement: Supplementary file 13 — Unprocessed WBs. [file 41564_2025_2068_MOESM13_ESM.pdf]
