# Supplementary material for: Multi-proteomic profiling of the varicella-zoster virus–host interface reveals host susceptibilities to severe infection
Source: Nat Microbiol. 2025 Jul 30;10(8):2048–72. doi: 10.1038/s41564-025-02068-7 (PMC12313529; doi:10.1038/s41564-025-02068-7)

Source Data 1 - Extended Data Fig. 2

Uncropped blots - Extended Data Fig. 2a

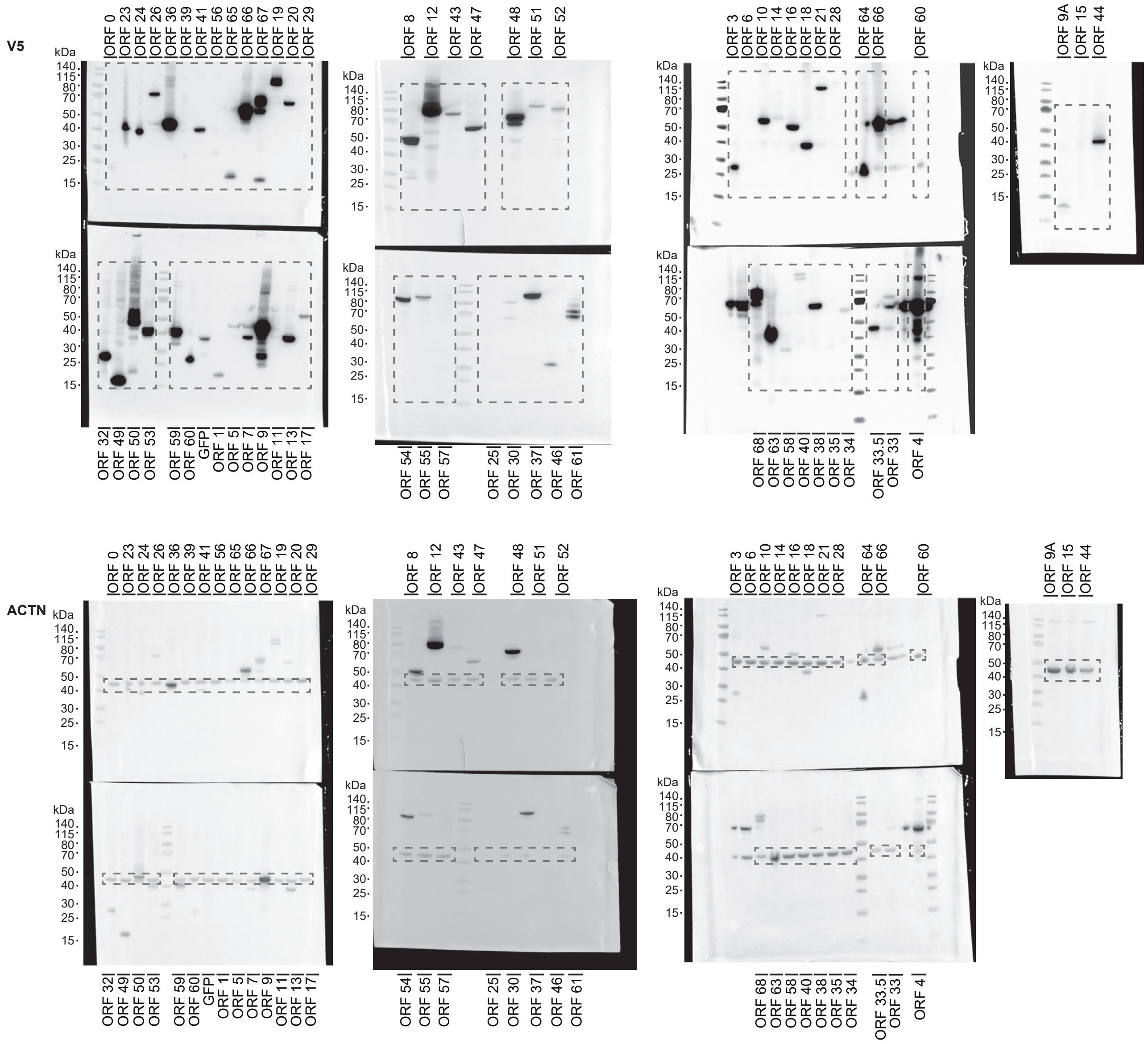

Uncropped blots - Extended Data Fig. 2f

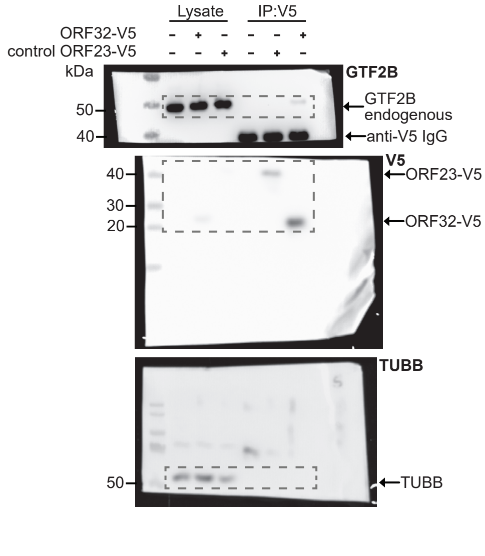

Uncropped blots - Extended Data Fig. 2g

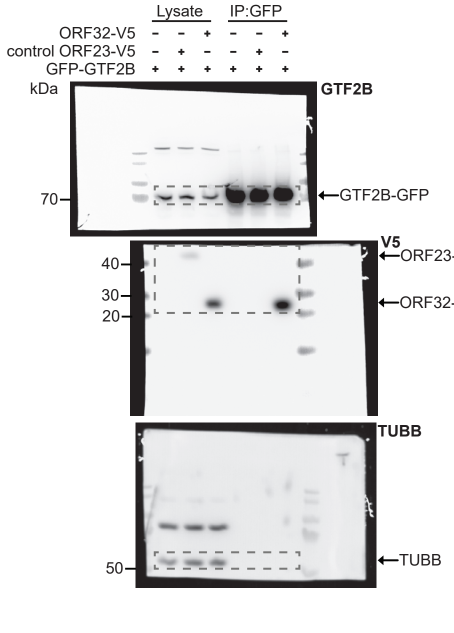

Supplement: Supplementary file 14 — Unprocessed WBs. [file 41564_2025_2068_MOESM14_ESM.pdf]
