# Supplementary material for: Multi-proteomic profiling of the varicella-zoster virus–host interface reveals host susceptibilities to severe infection
Source: Nat Microbiol. 2025 Jul 30;10(8):2048–72. doi: 10.1038/s41564-025-02068-7 (PMC12313529; doi:10.1038/s41564-025-02068-7)

Source Data 3 - Extended Data Fig. 6

Uncropped blot - Extended Data Fig. 6d

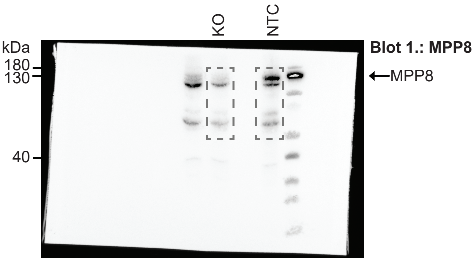

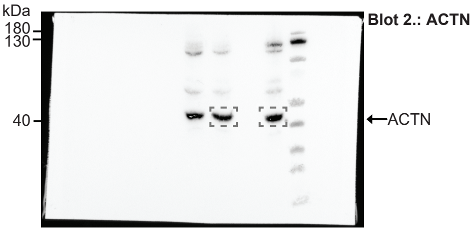

Uncropped blot - Extended Data Fig. 6j

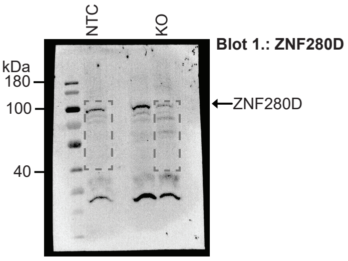

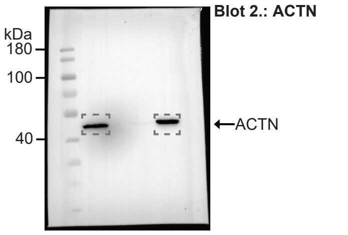

Supplement: Supplementary file 15 — Unprocessed WBs. [file 41564_2025_2068_MOESM15_ESM.pdf]
